# Contrasting Patterns of Rapid Molecular Evolution within the *p53* Network across Mammal and Sauropsid Lineages

**DOI:** 10.1093/gbe/evy273

**Published:** 2019-01-21

**Authors:** Courtney N Passow, Anne M Bronikowski, Heath Blackmon, Shikha Parsai, Tonia S Schwartz, Suzanne E McGaugh

**Affiliations:** 1 Department of Ecology, Evolution, and Behavior, University of Minnesota; 2 Department of Ecology, Evolution, and Organismal Biology, Iowa State University; 3 Department of Biology, Texas A&M University, College Station, TX; 4 Department of Biological Sciences, Auburn University, Auburn, AL

**Keywords:** *p53*-signaling network, lifespan, divergent molecular evolution, positive selection, mammals, sauropsids

## Abstract

Cancer is a threat to multicellular organisms, yet the molecular evolution of pathways that prevent the accumulation of genetic damage has been largely unexplored. The *p53* network regulates how cells respond to DNA-damaging stressors. We know little about *p53* network molecular evolution as a whole. In this study, we performed comparative genetic analyses of the *p53* network to quantify the number of genes within the network that are rapidly evolving and constrained, and the association between lifespan and the patterns of evolution. Based on our previous published data set, we used genomes and transcriptomes of 34 sauropsids and 32 mammals to analyze the molecular evolution of 45 genes within the *p53* network. We found that genes in the network exhibited evidence of positive selection and divergent molecular evolution in mammals and sauropsids. Specifically, we found more evidence of positive selection in sauropsids than mammals, indicating that sauropsids have different targets of selection. In sauropsids, more genes upstream in the network exhibited positive selection, and this observation is driven by positive selection in squamates, which is consistent with previous work showing rapid divergence and adaptation of metabolic and stress pathways in this group. Finally, we identified a negative correlation between maximum lifespan and the number of genes with evidence of divergent molecular evolution, indicating that species with longer lifespans likely experienced less variation in selection across the network. In summary, our study offers evidence that comparative genomic approaches can provide insights into how molecular networks have evolved across diverse species.

## Introduction

Cancer is a survival threat to most multicellular organisms. This strong selective pressure has given rise to mechanisms across diverse taxa that result in cancer prevention and suppression (Tollis, Schiffman, et al. 2017), including DNA repair, cellular apoptosis, and immune defenses against aberrant cells (Tollis, Boddy, et al. 2017). As cancer is generally caused by the accumulation of mutations within the cell, it is thought that if an organism has more cells (i.e., larger body size) and extended lifespan, then the incidence of cancer will be higher (Peto et al. 1975). Nonetheless, to date, there appears to be no correlation between the incidences of cancer with body size and/or longevity across species, known as Peto’s Paradox (Caulin and Maley 2011). Although cancer research in the past decade has begun including a broader range of taxa (Nagy et al. 2007; Abegglen et al. 2015; Nunney et al. 2015), the molecular evolution of genetic networks that prevent and repair the genetic damage spurring oncogenesis has been largely unexplored (Keane et al. 2015). One such network is the *p53*-signaling network, which has been linked to cancer for decades (Muller and Vousden 2014).

The tumor suppression gene *p53*, often termed the “guardian of the genome,” encodes transcription factor p53 that stabilizes the genome by regulating DNA-damage responses and cell fate decisions in response to DNA damage and stress (Levine and Oren 2009). Altered transcription of *p53* in response to such stress allows p53 to direct one of three responses: DNA repair, cell senescence, or cell apoptosis (Tyner et al. 2002; Reinhardt and Schumacher 2012). The *p53* gene along with the multitude of genes that either regulate *p53* expression or that are regulated by transcription factor p53 is best envisioned as a molecular network with *p53* as a central node (Matheu et al. 2008). Extensive research has identified hundreds of genes directly and/or indirectly associated with the *p53* network that can respond to and regulate DNA damage—with the consequence of tumor suppression (Levine et al. 2006).

**Fig. 1.**
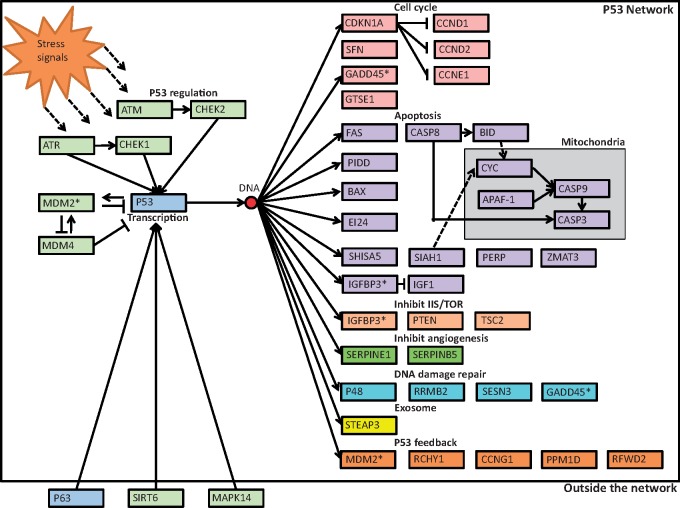
—Visualization of the *p53*-signaling network modified from the KEGG pathway. Included in this figure are the 45 proteins used in this study. These are 42 of 58 in the KEGG p53 pathway (Ogata et al. 1999) plus three genes (*p63*, *sirt6*, and *mapk14*) that are not in the KEGG *p53*-signaling network but are associated with the *p53* gene and were included as “outside the *p53* network.” For simplicity, we use the short-hand “*p53* network” within the text to refer to all of these 45 focal genes analyzed. Arrows after DNA correspond to all “downstream genes” in the network, whereas we considered genes upstream of this point to be “upstream genes.” Each color corresponds to the functional categories; green corresponds to genes associated with p53 regulation, blue are transcription factors, pink are genes involved with cell cycle, purple apoptosis, and light orange inhibit IIS/TOR, dark green inhibit angiogenesis, teal DNA-damage repair, yellow exosome, and orange p53 feedback. An asterisk next to a gene in the network gene indicates that the gene is part of multiple functional classifications (based on the KEGG *p53* pathway; Ogata et al. 1999).

The *p53* network has also been studied for its role in senescence—that is, declining function (such as pulmonary, cardiac, and aerobic), and increasing incidences of disease (e.g., cognitive impairment, hypertension, osteoporosis, Alzheimer’s, and cancer) that cause increasing mortality with advancing age. The *p53* network impacts senescence, both indirectly through its interaction with the insulin, insulin-like signaling (IIS) and Target-of-rapamycin (TOR) pathways (see fig. 1), and directly. Indeed, *p53* is of great interest to evolutionary biologists because it can function as an antagonistically pleiotropic gene (Ungewitter and Scrable 2009)—with beneficial effects early in life (i.e., tumor suppression) and detrimental effects later in life (i.e., the accumulation of senescent cells) (Hasty et al. 2016). For example, increased *p53* expression in two *p53* model systems resulted in increased tumor suppression but an overall decrease in longevity (Tyner et al. 2002; Maier et al. 2004). Thus, genes in the p53 family of transcription factors have been extensively studied in both cancer biology (Wasylishen and Lozano 2016) and aging biology (Wiley and Campisi 2016).

Despite the intensive study of this network, we still know very little about the evolution of the *p53* network. For example, although studies have observed reduced longevity with an increased expression of *p53* (Tyner et al. 2002; Maier et al. 2004), the effects are not attributable solely to the *p53* gene, but may also involve other genes in the *p53* network that modify p53 activity (Kanfi et al. 2012). Past studies on the evolution of the *p53* network have focused on only a handful of organisms (Reinhardt and Schumacher 2012) and have failed to leverage the striking diversity present in cancer incidence, physiology, and senescence across amniotes (mammals and sauropsids, which is defined as avian and nonavian reptiles) (see also Schiffman et al. 2015). Amniotes have evolved extreme metabolic and physiological plasticity in response to environmental stimuli (Schwartz and Bronikowski 2011; van Breukelen and Martin 2015). Relative to mammals, reptiles and birds have substantial diversity in body temperature and metabolic rate across the sauropsid clade, from high body temperature and metabolic rate in endothermic birds to fluctuating body temperature and metabolic rates in ectothermic reptiles (e.g., Gangloff et al. 2016). Temperature has long been associated with mutation rate (Muller 1928), hence, metabolic rate may affect mutation rates and therefore molecular evolution (Gillooly et al. 2005). Variation in body temperature and subsequent metabolic rate could impose diverse selection pressure on mutation repair mechanisms (e.g., the *p53* network) to compensate for variation in mutation rates across sauropsids more so than in mammals. Beyond these considerations of temperature, metabolic, and mutation rates, amniote lineages demonstrate great diversity in maximum lifespan (Jones et al. 2014) many with correlated life history traits (growth, maturation, and reproduction (Ricklefs 2010)). These life history traits may correlate with mechanisms for protection against and repair of DNA damage (e.g., Robert and Bronikowski 2010). Likewise, necropsy data suggest that sauropsids, including birds, exhibit overall lower cancer rates than mammals (Effron et al. 1977). However, within sauropsids, there is notable variation among lineages in cancer incidence with crocodilians having the lowest and squamates having the highest incidences (Garner et al. 2004). Similarly in mammals, variation in cancer incidence ranges from extremely low in naked mole-rats (Buffenstein 2005) and elephants (Abegglen et al. 2015) to very high in wild-type and transgene mice (Bult et al. 2015) and humans (Albuquerque et al. 2018).

To address the lack of taxonomically broad studies in our knowledge of the evolution of the *p53* network, we performed comparative genomic analyses of this network within and between the two lineages of amniotes—mammals and sauropsids. Sixty-six species were selected based on a previous study on the molecular evolution of IIS/TOR network (McGaugh et al. 2015) to allow for subsequent comparisons. Our overall objectives were to quantify the evolutionary constraints and hot-spots within the *p53* network. Such a comparative framework is necessary to understand whether sauropsids and mammals employ unique or shared evolutionary responses to stressors that cause DNA damage and that ultimately contribute to tumorigenesis. We used available genomes and transcriptomes from NCBI/Ensembl across amniotes along with additional transcriptomes that we previously generated (McGaugh et al. 2015) to analyze the molecular evolution of Kyoto Encyclopedia of Genes and Genomes (KEGG) *p53* network genes (Ogata et al. 1999). We included three additional genes that interact with the *p53* network; *sirt6* (Van Meter et al. 2011), *mapk14* (Fiordaliso et al. 2001), and *p63* (Dötsch et al. 2010) (fig. 1).

Throughout, we are interested in two aspects of molecular evolution. First, whether subsets of codons in protein-coding genes are experiencing positive selection in particular lineages (hereafter “positive selection”). Second, whether specific codons in protein-coding regions are experiencing different selection pressures in different taxa (e.g., codon-specific selective constraints that differ between mammalian and sauropsid clades—hereafter “divergent molecular evolution”). Thus, we specifically tested the following three questions. 1) Do mammals or sauropsids exhibit more evidence of either of these two aspects of molecular evolution in the *p53* network? 2) Do upstream genes evolve more quickly than downstream genes in the *p53* network both within and between mammals and sauropsids? Genes upstream in a network can control flux to the downstream genes (Wright and Rausher 2010), subjecting upstream genes to greater selective constraints and more conservation (Rausher et al. 1999). On the other hand, upstream genes may evolve more rapidly, potentially due to an increased number of interactions, and thus more pleiotropy, as compared with downstream genes (Alvarez-Ponce et al. 2011). 3) Is there an association between species-specific lifespan and the number of genes under selection in the *p53* network (i.e., both the number of genes with evidence of positive selection and the number of genes with evidence of divergent molecular evolution) such as has been reported in other taxa (naked mole-rat: Kim et al. 2011)?

We found that genes in the *p53* network exhibited evidence of extensive positive selection and divergent molecular evolution in mammals and sauropsids. Specifically, when we tested for lineage-specific selection, we found more genes with evidence of positive selection in sauropsid lineages as compared with mammalian lineages, suggesting that mammals and sauropsids have different targets for selection within the *p53* network. We also found substantial evidence of divergent molecular evolution between mammals and sauropsids, suggesting that the strengths and modes of selection have differed within the *p53* network, and that the *p53* network is exceptionally divergent relative to a proxy for the remainder of the genome. Moreover, our data suggest that for sauropsids, particularly squamates (snakes and lizards), the genes at the top of the regulatory network are likely the targets of recent selective forces compared with mammals. Lastly, we found a negative correlation between the maximum lifespan of a lineage and the number of genes in the network with divergent molecular evolution for that particular lineage, suggesting that species characterized by shorter lifespans have experienced modes of selection across their *p53* networks that are divergent from the remainder of the tree.

## Materials and Methods

### Identifying Candidate Orthologs and Generating Alignments and Gene Annotations

**Fig. 2.**
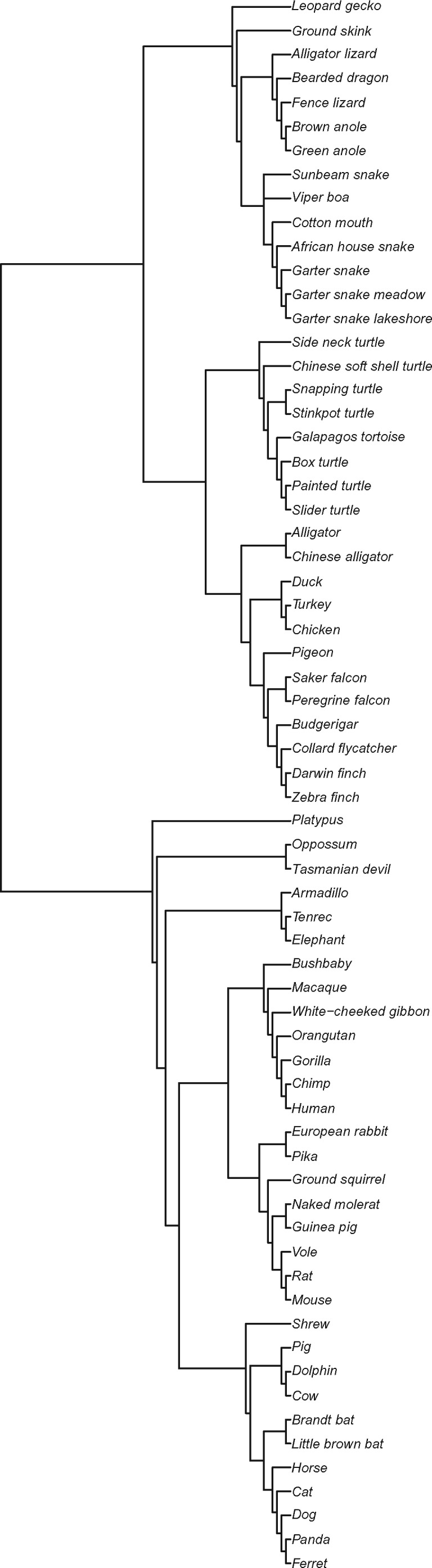
—Rooted cladogram. The cladogram is based on a previous published study (McGaugh et al. 2015) showing the phylogenetic relationships among all the species (both mammals and sauropids) included in this study. Analyses were conducted in PAML with an unrooted cladogram except where otherwise noted.

To identify genes in the *p53* network, we utilized our published data set of amniotes, which used 32 mammalian and 34 sauropsid (including 10 bird; fig. 2 and supplementary table S1, Supplementary Material online; McGaugh et al. 2015) genomes and transcriptomes from GenBank (Sequence Read Archive Study accessions: SRA062458 and SRP017466). From these, we were able to extract sequences for 42 of the 58 genes located upstream and downstream in the KEGG *p53* network (Ogata et al. 1999), plus three additional genes related to the *p53* network (*p63*, *mapk14*, and *sirt6*) as noted above, for a total of 45 orthologs (table 1 and supplementary table S2, Supplementary Material online). We employed similar methods as in a previous study (McGaugh et al. 2015) to curate this total of 45 orthologs in up to the 66 species. Because the branch leading to the common ancestor of mammals is the same as the common ancestor for sauropids, we also performed analyses with *Xenopus tropicalis* (frog) included as an outgroup to help polarize derived changes among the lineages of amniotes. This did not significantly alter the overall conclusions regarding evolution of this network; some individual genes differed in their significance level between analyses that included and excluded the frog genome (see supplementary material, Supplementary Material online, for details on methods and results). Thus, we focused on the analysis without frog for the majority of this work.

**Table 1:** *p53* Network Genes, Gene Names, and Number of Focal Species Used for Each Gene

Gene	Functional Class	Protein Description	Focal Number of Species
ATM	p53 regulation	ATM serine/threonine kinase	64
ATR	p53 regulation	ATR serine/threonine kinase	65
MAPK14	p53 regulation	Mitogen-activated protein kinase 14	58
CHEK1	p53 regulation	Checkpoint kinase 1	62
CHEK2	p53 regulation	Checkpoint kinase 2	51
MDM2	p53 regulation/p53 feedback	MDM2 proto-oncogene, E3 ubiquitin protein ligase	66
MDM4	p53 regulation	MDM4, p53 regulator	63
SIRT6	p53 regulation	Sirtuin 6	60
P53	Transcription	Tumor protein p53	52
P63	Transcription	Tumor protein p63	47
CDKN1A	Cell cycle	Cyclin-dependent kinase inhibitor 1A (p21, Cip1)	62
CCND1	Cell cycle	Cyclin D1	62
CCND2	Cell cycle	Cyclin D2	52
CCNE1	Cell cycle	Cyclin E1	58
SFN	Cell cycle	Stratifin	45
GADD45G	Cell cycle /DNA-damage repair	Growth arrest and DNA-damage-inducible, gamma	48
GTSE1	Cell cycle	G-2 and S-phase expressed 1	49
FAS	Apoptosis	Fas cell surface death receptor	54
CASP8	Apoptosis	Caspase 8, apoptosis-related cysteine peptidase	50
BID	Apoptosis	BH3 interacting domain death agonist	62
PIDD	Apoptosis	P53-induced death domain protein 1	53
BAX	Apoptosis	BCL2-associated X protein	46
EI24	Apoptosis	Etoposide induced 2.4	66
SHISA5	Apoptosis	Shisa family member 5	59
PERP	Apoptosis	PERP, TP53 apoptosis effector	64
ZMAT3	Apoptosis	Zing finger, matrin-type 3	53
SIAH1	Apoptosis	Siah E3 ubiquitin protein ligase 1	64
CYC	Apoptosis	Cytochrome c, somatic	59
APAF1	Apoptosis	Apoptotic peptidase activating factor 1	64
CASP9	Apoptosis	Caspase 9, apoptosis-related cysteine peptidase	50
CASP3	Apoptosis	Caspase 3, apoptosis-related cysteine peptidase	60
IGFBP3	Apoptosis/inhibit IIS/TOR	Insulin-like growth factor binding protein 3	58
IGF1	Inhibit IIS/TOR	Insulin-like growth factor 1	58
PTEN	Inhibit IIS/TOR	Phosphatase and tensin homolog	66
TSC2	Inhibit IIS/TOR	Tuberous sclerosis 2	66
SERPINE1	Inhibit angiogenesis	Serpin peptidase inhibitor, clade E (nexin, plasminogen activator inhibitor type 1)	49
SERPINb5	Inhibit angiogenesis	Serpin peptidase inhibitor, clade B (ovalbumin), member 5	47
P48 (DDB2)	DNA-damage repair	Damage-specific DNA binding protein 2	62
RRM2b	DNA-damage repair	Ribonucleotide reductase M2 B (TP53 inducible)	58
SESN3	DNA-damage repair	Sestrin 3	62
STEAP3	Exosome	STEAP family member 3, metalloreductase	64
RFWD2	p53 feedback	Ring finger and WD repeat domain 2, E3 ubiquitin protein ligase	66
RCHY1	p53 feedback	Ring finger and CHY zinc finger domain containing 1, E3 ubiquitin protein ligase	52
CCNG1	p53 feedback	Cyclin G1	62
PPM1D	p53 feedback	Protein phosphatase, Mg^2+^ Mn^2+^ dependent, 1D	65

Note.—Reported are the gene names (symbols are HGNC gene symbols), functional classes, and protein descriptions of the 45 genes analyzed here that are associated with the *p53* pathway. We also report the number of focal species used for each gene (out of the total 66 species).

For all alignment and gene annotation analyses performed, we used the data sets generated from McGaugh et al. (2015) and followed the same methods detailed in that work. In the original alignments, we started with 74 species, which represented extensive data mining at the time these data sets were created, including caiman, loggerhead sea turtle, corn snake, European pond turtle, Hilaire’s side-necked turtle, python, quail, and tuatara. These eight species were removed then and in this analysis because data available at the time were preliminary and dramatically reduced the possible number of ortholog alignments. In brief, transcriptome-derived open reading frames and genome-derived gene sequences were clustered with USEARCH to reduce redundancy among isoforms, followed by clustering with OrthoMCL (Li et al. 2003). Multiple sequences per species were often present in the OrthoMCL clusters. To address this, we used USEARCH to identify clusters of sequences (Edgar 2010), within the OrthoMCL clusters. If a single species still had multiple sequences in the USEARCH cluster, we used the sequence from each species that was closest to the centroid identified for that particular cluster by USEARCH (see supplementary material, Supplementary Material online).

Within the *p53* network, 12 of the 45 genes were split among separate USEARCH clusters that were often taxon-specific clusters (e.g., a sauropsid cluster and a mammal cluster). Thus, clusters for each of these 12 genes were combined post-USEARCH and realigned into a single alignment per gene with only a single sequence representing each species (the longest was chosen if there were multiple sequences per species after combining clusters, see supplementary material, Supplementary Material online, for details). Amino acid alignments were performed with MSAProbs (Liu et al. 2010). Alignments were back-translated using the MSAProbs amino acid alignments and the original nucleotide sequences using RevTrans (Wernersson and Pedersen 2003). The command-line version of Translator X was used with the MSAProbs amino acid alignments to produce GBlocks-cleaned amino acid and nucleotide alignments (Talavera and Castresana 2007; Abascal et al. 2010). Alignments for focal genes were manually corrected for misaligned indels, which usually occurred near the ends of the sequences.

To annotate and curate a focal gene set for the *p53* network, we used BlastP version 2.2.28 (Altschul et al. 1990) to identify the best match for every sequence in each alignment using the UniProt database as the BLAST database. We made a separate BLAST database of KEGG pathway p53 network proteins from chicken or anole (Kanehisa and Goto 2000). We classified our annotation as correct if both the UniProt and KEGG database BLAST searches resulted in identical best blast hits. If paralogs were found through this method, we excluded that particular sequence and realigned using procedures described above.

From our original alignments, we identified 1,414 genes to serve as a proxy for the remainder of the genome and we refer to these as “control” genes. The control genes contained sequences for each of the 66 species. We included only genes that contained the total set of 66 species in the control gene set as a complete phylogenetic tree was the most efficient input for phylogenetic analysis by maximum likelihood (PAML) for our control genes, and this option did not require deleting taxa, remaking trees, and redesignating PAML foreground branches for each of the 1,414 genes.

### Statistical Tests of Molecular Evolution

To identify evidence of positive selection and divergent selection histories between mammals and sauropsids in *p53* network genes, we used the codeml program in PAML version 4.7 (Yang 2007). We used the phylogenetic tree constructed previously (McGaugh et al. 2015). In brief, to construct the tree, we combined results from previous studies (Hedges and Kumar 2009; Thomson and Shaffer 2010; Perelman et al. 2011; dos Reis et al. 2012; Wiens et al. 2012; Kimball et al. 2013; McCormack et al. 2013) to generate a tree topology with no branch lengths. Newick Utilities was used to prune this base tree to remove any taxa that were missing in the alignment for each focal gene (Junier and Zdobnov 2010). For analyses that required branch lengths, we used median dates from TimeTree (Hedges et al. 2006; Kumar et al. 2017). We used PAML to calculate omega (*ω*), which is defined as the rate of nonsynonymous substitutions per nonsynonymous sites (d*N*) over the rate of synonymous substitutions per synonymous sites (d*S*) in a protein-coding sequence (Goldman and Yang 1994).

To assess the probability that specific genes on a branch of the tree experienced positive selection, branch-site models were applied to each gene individually (Zhang et al. 2005). Branch-site models test whether specific user-chosen “foreground branches” exhibit a different *ω* from background branches (i.e., the remainder of the tree). Specifically, the branch-site test compares a model with a subset of positively selected sites in the foreground branch/clade (Yang 2007) versus a model where *ω* is fixed and equal to one (null model) using a Likelihood Ratio Test (LRT). For each LRT, the test statistic was compared with a 1:1 mixture of *χ^2^* distributions with 1 and 0 degrees of freedom (Goldman and Whelan 2002). *P* values were corrected for multiple tests via sequential Bonferroni (Holm 1979), though using false discovery rate correction (FDR) produced nearly identical results (data not shown). Sequential Bonferroni methods were used, as standard Bonferroni correction may be overly conservative. We performed a separate sequential correction for each branch-site test.

For each gene in the *p53* network, we first set either the ancestral sauropsid branch or the entire sauropsid clade in the foreground branch. We then repeated this with either the ancestral mammal branch or the entire mammal clade as the foreground branch. In addition, we performed separate tests setting the foreground branch as the branch leading to squamates (lizards and snakes combined), lizards, snakes, turtles, crocodilians, and birds for testing within sauropsids as well as primates, rodents, marsupials, bats, and monotremes for testing within mammals. We focused on specific sauropsid and mammalian groups where previous research that quantified variation in either the p53 gene and/or network (e.g., Seim et al. 2013; Abegglen et al. 2015; Alibardi 2016) or where species exhibited notable lifespan differences (Kim et al. 2011). For example, we included a test of the branch leading to elephants as substantial research has been done on copy number variation in *p53* in elephants, and they are a long lived species with very few mutations in *p53* (Abegglen et al. 2015; Sulak et al. 2016). Bayes Empirical Bayes output was used to identify the specific sites with strong evidence of positive selection.

We used clade model C (Bielawski and Yang 2004) to test for divergent molecular evolution (i.e., evidence that *ω* in a focal clade differed from *ω* estimated from the rest of the tree [Yang and Bielawski 2000]). Unlike the branch-site test that identifies evidence of positive selection, the clade model tests for divergent *ω* between clades but does not constrain the *ω* to be >1. For the clade models, we tested entire clades and not ancestral branches leading to particular clades. For the null hypothesis, we used the M2a_rel model (Weadick and Chang 2012). Significance was assessed via a LRT between the null (no difference in *ω* between two clades) and alternative models (differences in *ω* between the test clade and the remainder of the tree). *P* values were adjusted with sequential Bonferroni (Holm 1979) as described above. For all focal genes that were significant via the clade model, we compared the *ω* values (i.e., d*N*/d*S*) for each clade via paired Wilcoxon tests and *χ*^2^ tests.

### Network Location Effects on Molecular Evolution

We utilized *χ*^2^ tests to determine whether the number of genes in the network identified with evidence of positive selection (branch-site tests) or divergent molecular evolution (clade model C) differed within upstream and downstream genes, and within or among clades. For this analysis, genes are classified as “upstream” or “downstream” based on their direct or indirect interaction with p53 (fig. 1). Therefore, genes outside the *p53* network (*sir6*, *mapk14*, and *p63*) were included with upstream genes because they interact with p53. Because sample sizes on these *χ*^2^ tests are often small we calculated *P* values using 2000 Monte Carlo simulations (Hope 1968).

### Measures of Lifespan

Species-specific maximum lifespan data were downloaded from the AnAge database (Tacutu et al. 2012). If no data were available for a species, we performed a literature search to identify this species-specific maximum lifespan (supplementary table S1, Supplementary Material online). For focal groups that included more than one species, we defined maximum lifespan as the median of the distribution of species-specific maximum lifespans (see supplementary table S2, Supplementary Material online). To test for a relationship between a lineage’s maximum lifespan and the pattern of molecular evolution within the *p53* network, we performed linear regressions of the number of genes that were significant in each lineage (after sequential Bonferroni correction) in both species-specific branch-site and clade model tests on the mean of the maximum lifespans for species in each clade. We first performed a standard linear regression using the lm function in R. Then, to test whether a phylogenetic correction was necessary, we calculated the Blomberg *K* statistic based on the residuals of the standard regression using the R package Phytools (Blomberg et al. 2003; Revell 2012). To account for phylogeny, we used the tree with branch lengths (described above) and the generalized least squares (GLS) function from the R package nlme and specified a correlation structure using the corBrownian function from the R package ape (Paradis et al. 2004; Pinheiro et al. 2014). Exploratory analyses indicated that results from standard and phylogenetically corrected regressions were quantitatively different but qualitatively similar. All tests were run with an alpha level of 0.05.

## Results

### *p53* Network Alignments

We created alignments for 45 genes within the *p53* network (fig. 1 and table 1). The number of species ranged between 45 and 66 per alignment (mean = 57.8, median = 59, and mode = 62; supplementary table S2, Supplementary Material online). Of the 45 genes, we obtained sufficient species coverage of genes upstream and downstream (genes upstream and including *p53* and *mdm2 *=* *10 and genes downstream *p53* in the network = 35).

### Unique Evolutionary Rates in the *p53* Network between Sauropsids and Mammals

We performed pairwise d*N*/d*S* comparisons first to quantify the difference in evolutionary rates between the p53 network genes in sauropsids and mammals compared with a control set of genes. Of the 45 genes in the *p53* network with sufficient numbers of species (*N* ≥ 45), 12 (*bax*, *bid*, *casp8*, *cdkn1a*, *fas*, *gtse1*, *mdm2*, *p48*, *p53*, *perp*, *serpine1*, and *shisa5*) were split among multiple USEARCH clusters (see Materials and Methods) and were combined post hoc and then realigned. In many cases, these genes were split into taxonomic clusters (e.g., the sauropsids were split from the mammals), supporting that they were likely exceptionally divergent genes.

As expected, we found that these split and post hoc combined genes were more divergent as compared with the remainder of the genes in the network that had a single dominant cluster per gene (median combined ***ω*** = 0.22, median not combined ***ω*** = 0.07, Kruskal–Wallis = 17.67, df = 1, *P* < 0.01, supplementary table S3, Supplementary Material online). Because each control gene was derived from a single dominant cluster, we limited our d*N*/d*S* comparisons with these 1,414 control genes to the 33 (out of 45) *p53* network genes that formed a single dominant cluster (i.e., excluding the 12 noted above). For each gene, we used the median of all pairwise d*N*/d*S* measures between each sauropsid and mammal (see supplemental material) and found that genes in the *p53* network exhibited larger d*N*/d*S* values between sauropsids and mammals than control genes (e.g., a proxy for rest of the genome). When examining the top 5% of d*N*/d*S* median values among the 1,414 control + 33 focal genes, 8 genes from the focal gene set appeared in the top 5% (odds ratio 6.75 [95% CI 2.93, 15.55]), indicating that focal genes were approximatively 7 times more likely to exhibit d*N*/d*S* in the top 5% compared with the control set (supplementary table S4, Supplementary Material online). Thus, even when we excluded the 12 most divergent *p53* network genes, the remaining *p53* network genes contained faster evolving components as compared with a proxy for the remainder of the genome, suggesting this network has been strongly selected on during the evolution of sauropsids and mammals.

### Positive Selection in the *p53* Network Using Branch-Site Models

Because the evolutionary rate of the p53 network had faster evolving components compared with the background set, we examined genes with evidence of positive selection between mammals and sauropsids using species-specific branch-site models. Within the *p53* network, a total of 31 out of 45 genes were significant after Bonferroni correction in at least one branch-site test for positive selection (total genes in supplementary tables S5–S7, Supplementary Material online, that are bold face with asterisks), and a substantial proportion of the network exhibited evidence for positive selection along the branches leading to mammals and/or sauropsids (supplementary tables S6 and S7, Supplementary Material online), indicating that these changes may have been important in the evolution of the two major amniote groups.

**Fig. 3.**
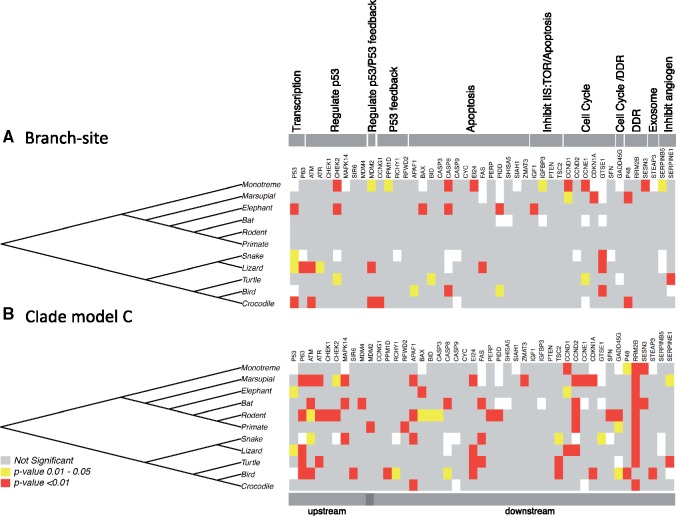
—Phylogenetic heat maps for significant genes in the *p53* network. Depicted are heat maps for (*A*) branch-site tests and (*B*) clade model C tests. Visualization of the *p53* genes that were significant for positive selection (branch-site) or divergent molecular evolution (clade model C) based on sequential Bonferroni corrected *P* values. In the *p53* network, genes were grouped based on functional classification and whether they were upstream or downstream in the network. If a gene is colored white, then there was no sequence available for that group.

Individual lineages exhibited evidence of positive selection throughout the *p53* network (fig. 3*A* and supplementary tables S6 and S7, Supplementary Material online). For example, after correction for multiple testing, all individually examined lineages of sauropsids exhibited evidence of genes under positive selection (fig. 3*A*). In contrast to sauropsids, several mammalian lineages exhibited no positive selection within the p53 network (rodents, primates, and bats after multiple testing corrections [supplementary tables S6 and S7, Supplementary Material online]). Marsupials, the monotreme, and the elephant were the only tested lineages of mammals that exhibited evidence of positive selection (fig. 3*A* and supplementary table S6, Supplementary Material online). Results were consistent from alignments containing frog except one gene in primates exhibited evidence of positive selection (supplementary tables S6 and S7, Supplementary Material online). Therefore, within the major clades, selection on this network is more concentrated in sauropsids, particularly in squamates (fig. 3*A* and supplementary tables S6 and S7, Supplementary Material online).

Because within the *p53* gene itself there were multiple codons with evidence of positive selection, we mapped the amino acid residues onto the human p53 protein reference sequence (supplementary fig. S1*A*, Supplementary Material online). For the branch-site test of the mammalian ancestral branch, we identified one site in the p53 DNA binding domain with evidence of positive selection (100Q in most mammals → P in *Chiroptera* and *Caniformia*, H in *Hystricomorpha* [guinea pigs and naked mole-rat] and T in sauropsids). For the mammalian clade, two sites in the tetramerization domain—a domain necessary for DNA binding and other functions (Chene 2001)—had evidence of positive selection: 337R in most mammals → N in guinea pig and Chinese softshell turtle, and R in most squamates; and site 342R in most mammals → L in elephant, W in shrew, S in vole, Q in squirrel, and K in all sauropsids. When elephant was set in the foreground, four sites within a six amino acid window were found to have evidence positive selection (supplementary fig. S1*B*, Supplementary Material online). Although the function of these changes is not evident, they are located in the DNA binding domain and within the region that has been documented to interact with the following proteins: AXIN1, HIPK1, FBXO42, CCAR2, and ZNF385A (supplementary fig. S1*B*, Supplementary Material online). When the branch leading to sauropsids was in the foreground, we identified one site in the nuclear export signal domain of p53 with evidence of positive selection (345N in all mammals, turtles, and crocodilians → L in most squamates except for R in alligator lizard). This same substitution was significant for positive selection both when the branch leading to squamates (lizards and snakes) and the sauropsid clade were placed in the foreground. Indeed, when the sauropsid clade was placed in the foreground, a total of 25 sites (including 345N) were significant for positive selection. Finally, there were four sites with evidence of positive selection within lizards specifically, two of which were in the bipartite nuclear localization signal domain. This analysis suggests that the *p53* network, particularly *p53* itself has been a target of selection throughout amniote evolution, and these amino acid changes may provide interesting avenues for future work.

### *p* *53* Network Genes with Evidence of Divergent Molecular Evolution Based on Clade Model C

Clade models were used to test for divergent molecular evolutionary regimes in different clades of mammals and sauropsids relative to the rest of the tree. Note, clade models are less prone to false positives than branch-site models and better account for among-site variation in selective constraint (Weadick and Chang 2012). For both the sauropsid and mammalian clades, the molecular evolution for each clade is different relative to the remainder of the tree for 35–44% of the genes examined (supplementary tables S8 and S9, Supplementary Material online). In lineage-specific tests, squamates, birds, rodents, and marsupials exhibited the most genes experiencing divergent molecular evolution (fig. 3*B* and supplementary tables S8 and S9, Supplementary Material online). Approximately, one-third of the tested genes in these lineages exhibited evolutionary patterns that were significantly divergent from the rest of the tree.

### Enrichment of Significant Genes Located Upstream versus Downstream in the *p53* Network Is Driven by Sauropsids

We also tested whether genes upstream or downstream in the network evolve quicker within and between mammals and sauropsids. Hence, we tested for enrichment of genes that were significant for PAML tests based on their placement in the network using *χ*^2^ tests and Monte Carlo simulations. For the branch-site models, we found that sauropsids had a higher proportion of upstream relative to downstream genes under positive selection in the *p53* network (*χ^2^* = 6.2042; *P* value = 0.0245). In contrast, mammalian lineages exhibited no difference between upstream and downstream genes in the numbers that experienced positive selection (*χ^2^* = 0.017; *P* value = 1.0). In line with these results, sauropsids also had a significantly larger proportion of upstream genes under positive selection compared with upstream genes in mammals (*χ^2^* = 4.6459; *P* value = 0.0465), but no difference was present between sauropsids and mammals in the proportion of downstream genes under selection (*χ^2^* = 0.1125; *P* value = 0.8336).

Because more genes with evidence of positive selection were upstream in the network for sauropsids, we tested whether a specific lineage was driving this pattern. We used Monte Carlo simulations to test for an overabundance of genes experiencing positive selection upstream or downstream in the network for each sauropsid lineage tested. We found that only squamates exhibited significant enrichment for upstream genes compared with mammals (*χ^2^* = 6.9605; *P* value = 0.0155), and this relationship is consistent for alignments including frog (*χ^2^* = 4.150; *P* value = 0.046; supplementary tables S10 and S11, Supplementary Material online). Note, when frog is included, we also see a significant enrichment of positively selected downstream genes in mammals relative to sauropsids (*χ^2^* = 4.306; *P* value = 0.047; supplementary table S11, Supplementary Material online), however this relationship is a weak trend and may be driven by the poor alignments (see supplementary material, Supplementary Material online).

Unlike the branch-site tests, for the clade models, we found no evidence that upstream versus downstream genes were subject to more divergent molecular evolution for any comparison (supplementary tables S10 and S11, Supplementary Material online). Therefore, our data suggest that network location is significantly associated with propensity to be a target of positive selection and squamates (lizards and snakes) are likely driving the enrichment of upstream genes under positive selection observed in sauropsids.

### Associations of Maximum Lifespan and Molecular Evolution in the *p53* Network

As many mammals and sauropsids demonstrate diversity in lifespan and incidences of cancer, we tested if there was an association between species-specific lifespan (supplementary table S1, Supplementary Material online) and the number of genes under selection within the *p53* network. Using the sequential Bonferroni corrected *P* values, we observed no significant correlation between maximum lifespan and the number of genes under positive selection in the network (*β* = 0.002, *R*^2^ = −0.11, *P* value = 0.96). When frog was included, we saw a weak, significant correlation between genes under positive selection in the network and maximum lifespan (*β = *0.056, *R*^2^ = 0.08, *P* value = 0.050, supplementary fig. S2, Supplementary Material online).

**Fig. 4.**
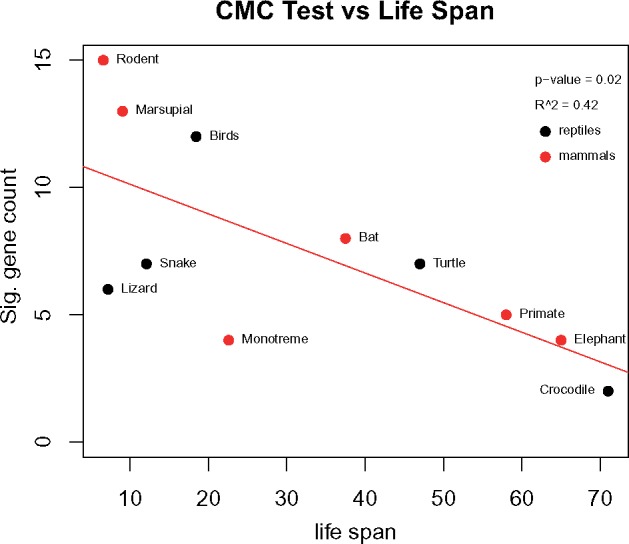
—Correlation between longevity and divergence in molecular evolution of the *p53* network. “Sig. gene counts” is the number of genes with significant evidence of divergent *ω* through clade model C after sequential Bonferroni corrections. Lifespan is based on the median of the maximum lifespans (see supplementary table S1, Supplementary Material online) for all species used in lineage-specific comparisons.

In contrast, for clade model C, we found a negative association of maximum lifespan with the number of genes experiencing divergent *ω* in respective clades. Longer-lived species have fewer genes with significantly different *ω* in the focal clade than in the remainder of the tree as compared with short-lived species (fig. 4; *β =−*0.116, *R*^2^ = 0.42, *P* value = 0.02). Likewise, when frog was included, we saw similar results, (supplementary fig. S3, Supplementary Material online; *β =−*0.09, *R*^2^ = 0.50, *P* value = 0.01). Notably, the clade model measures differences in *ω* between the foreground and background; hence, the genes could be under purifying or positive selection. Specifically, this negative correlation between maximum lifespan and the number of genes with divergent *ω* could be due to some foreground clades exhibiting more purifying selection than the remainder of the tree (*ω* foreground < *ω* background) and other foreground clades exhibiting more positive selection than the remainder of the tree (*ω* foreground > *ω* background). Therefore, we calculated a statistic Δ*ω*, which is the difference in *ω* calculated for the focal (foreground) taxa relative to the background taxa in each clade model. Generally, we interpret positive values of Δ*ω* to suggest more positive selection in the foreground taxa relative to the background taxa, whereas negative Δ*ω* values suggest purifying selection in the foreground taxa. We found that both positive and negative Δ*ω* scores drive significant clade models for taxa with short lifespans (supplementary fig. S4, Supplementary Material online). In sum, it appears that rodents and marsupials (i.e., animals with shorter lifespans) exhibit more genes with divergent evolutionary patterns relative to the rest of the tree than do organisms with longer lifespans (e.g., crocodilians, elephants, and primates).

One potential concern is that variation in lifespan within a clade strongly influences this result. For instance, primate maximum lifespans vary by as much as an order of magnitude. To explore the impact of this variation on our analysis, we also performed a weighted least squares regression where the weight placed on each lineage was equal to the inverse of the variance in lifespans in the lineage. The results from this weighted regression yielded regression coefficients and significances that were qualitatively similar to the unweighted analysis (supplementary table S12, Supplementary Material online).

## Discussion

The *p53* network prevents tumorigenesis and is a prominent focus of cancer biology (Agarwal et al. 1998). Past research has identified evidence of positive selection across genes and pathways associated with apoptosis and cancer in mammals (Crespi and Summers 2006; Kosiol et al. 2008; Gaur et al. 2017). Across diverse taxa however, very little is known about the evolution of the *p53* network and its association with cancer (Levine et al. 2006; Aktipis et al. 2015). Yet, there is widespread agreement that a comparative perspective on oncology, and the genes underlying cancer development, can provide insights into conserved and novel solutions to the problem of tumorigenesis across the tree of life (Tollis, Schiffman, et al. 2017). For example, a previous study found evidence that sauropsids have lower incidence of cancer than mammals (Effron et al. 1977). In mammals, elephants and naked mole-rats exhibit low cancer incidences (Buffenstein 2005; Abegglen et al. 2015). Thus, our wider taxonomic examination of the evolution of the *p53* network is relevant to an understanding of the genetics underlying variation in cancer prevalence across amniotes.

### *p53* Network Genes Are Outliers in Evolutionary Rates between Sauropsids and Mammals

Across mammal and sauropsid taxa, many evolutionary innovations have arisen in association with adapting to diverse conditions (Schwartz and Bronikowski 2011; van Breukelen and Martin 2015), including variation in body temperature and metabolism (Gangloff et al. 2016). In turn, these may have been facilitated by substantial molecular evolutionary shifts (Bromham 2011; McGaugh et al. 2015). In this study, we found that genes throughout the *p53* network exhibited divergence within and between the sauropsid and mammal clades. This finding is similar to our previous study demonstrating both sauropsids and mammals exhibited divergence in a large proportion of genes associated with IIS/TOR network, which regulates lifespan, reproduction, metabolic diseases, and cancer (McGaugh et al. 2015). Comparing d*N*/d*S* between *p53* network genes and a proxy for the rest of the genome (i.e., *p53* network genes = 33 and non-*p53* network genes = 1,414, see supplementary material, Supplementary Material online, for details), we found that the *p53* network genes are at a minimum 7 times more likely to be in the top 5% of d*N*/d*S* values compared with the proxy for the rest of the genome (supplementary table S4, Supplementary Material online). This finding is certainly a conservative estimate given that we removed from this comparison *p53* network genes whose great divergence precluded a single dominant cluster (*bax*, *bid*, *casp8*, *cdkn1a*, *fas*, *gtse1*, *mdm2*, *p48*, *p53*, *perp*, *serpine1*, and *shisa5*).

Like many comparative studies of this nature, much of our analysis is dependent on synonymous mutations being effectively neutral and nonsynonymous mutations having a fitness effect by changing an amino acid sequence. Such concerns are important to consider because divergences between different sauropsid lineages are much deeper in time than divergences between mammalian lineages, and these deeper divergences may be more susceptible to saturation in synonymous changes, among other impacts on our analyses. In addition, across such a broad sampling of taxa, and across geographic regions within sampled taxa, it is quite likely that effective population sizes vary greatly. If some lineages are characterized by strikingly smaller effective population size, then many amino acid changes may behave as neutral mutations (Ohta 1992). Conversely, in lineages with increased effective population size, some synonymous sites may be under selection for translational efficiency (Waldman et al. 2011). Any of these possibilities could lead to a bias in our estimate of d*N*/d*S* ratios and subsequent analyses described in this manuscript.

### Tumor Suppressor Gene *p53* Shows Evidence of Positive Selection

Remarkably, one of the most frequently significant genes was *p53* itself, particularly when frog was not included. We identified evidence of positive selection in the *p53* gene in mammal (elephants) and sauropsid (squamates and crocodiles) lineages, as well as the branch leading to all sauropsids (fig. 3*A* and supplementary table S6, Supplementary Material online). When frog was included, only elephants were significant for the branch-site test (supplementary table S7, Supplementary Material online). *p53* has been a major focus in human cancer research since the discovery of its association with tumor suppression three decades ago (Finlay et al. 1989). Tumor-associated mutations usually occur in the region of the *p53* gene that encodes the DNA binding domain of the protein, and ultimately inactivates the apoptotic function of p53 (Kruiswijk et al. 2015). Previous phylogenetic analysis of *p53* has uncovered positive selection that acted on residues influencing the binding of p53 to DNA in mammals (Pintus et al. 2007). Our results reveal that taxa with evidence of positive selection in the *p53* gene (supplementary fig. S1, Supplementary Material online) are those with some of the lowest incidences of cancer reported in amniotes (elephant: Abegglen et al. 2015; snakes and lizards: Effron et al. 1977; crocodiles and turtles: Garner et al. 2004).

### Positive Selection across Lineages for *p53* Network Genes

We found that nearly two-thirds of the 45 genes we evaluated in this network exhibited evidence of positive selection in at least one branch-site test (i.e., in at least one lineage), and 31–38% of all genes in the network were significant for branch-site tests in multiple taxonomic groups (fig. 3*A* and supplementary tables S6 and S7, Supplementary Material online). This is similar to a previous study focusing on six mammal genomes that found evidence of positive selection (albeit on different genes in the network) acting on the *p53* network (Kosiol et al. 2008). The groups with the most genes under positive selection consistently among analyses with and without frog were squamates for reptiles, and monotremes for mammals (supplementary tables S6 and S7, Supplementary Material online). Interestingly, the trend with squamates is similar to previous research on IIS/TOR (McGaugh et al. 2015), which is a network that interacts with the *p53* network. Many of the genes under positive selection directly interact with *p53*—either as regulators of *p53* in the upstream portion of the network (*chek2*, *mdm2*, and *atr*), or as targets of *p53* (*pidd* in an apoptosis pathway; *p48* in a DNA-damage-repair pathway). Whether this represents correlated evolutionary changes in these genes in concert with the specific amino acid changes that have accumulated in the *p53* gene across diverse lineages (supplementary fig. S1, Supplementary Material online) or independent molecular evolution will be an exciting future area of research.

More broadly, nonmodel organisms may reveal much about p53 and associated network genes. For example, in elephants, the duplicate gene *lif6* responds to DNA damage by inducing apoptosis and is upregulated by p53 (Vazquez et al. 2018). This gene is under positive selection in elephants and appears to be associated with reduction of cancer incidence despite increased body size in this lineage. We expect similar deep explorations into *p53* and associated genes in nonmodel systems will yield fruitful results.

### Divergent Molecular Evolution across Lineages for *p53* Network Genes

Most of the *p53* network genes we examined exhibited evidence of divergent molecular evolution in one or more lineages relative to the remainder of the tree using clade model C (fig. 3*B* and supplementary tables S8 and S9, Supplementary Material online). In practice, this means that for most genes, the specific evolutionary pressures each gene has experienced have varied widely across species— with rodents, marsupials, bats, and squamates exhibiting the highest number of significant genes.

Similar, to the branch-site test, we identified evidence for divergent molecular evolution for *p53* in squamates and the entire mammalian clade (fig. 3*B* and supplementary tables S8 and S9, Supplementary Material online), further underscoring our interpretation above that the *p53* gene has been a target of selection in many amniote species, but in ways that vary among species. Interestingly, we also found strong evidence for divergent molecular evolution across mammals (marsupials and rodents) and sauropsids (lizards, turtles, and birds) in *p53*’s homolog *p63* (fig. 3*B*). Although there has been substantial research on *p53* in the context of cancer suppression, *p63* is complex and has given rise to proteins that both functionally resemble and counteract p53, indicating that p63 may have different physiological functions in the p53 protein family that need to be further explored (Yang et al. 2002).

### Enrichment in Upstream Genes under Selection

Organization of genes in a molecular pathway can influence the impact that mutations might have on the target phenotype (Cork and Purugganan 2004). For example, more highly connected genes (e.g., core genes, which are defined by the number of other genes the core gene is directly connected with) (Hahn and Kern 2005) or genes at branch points in a pathway (Flowers et al. 2007) are expected to exhibit different evolutionary rates than the peripheral genes with fewer connections. In this study, we found that a higher number of genes upstream in the network had evidence of positive selection in sauropsids than mammals (supplementary tables S10 and S11, Supplementary Material online), which indicates that the earliest-acting genes are the predominant targets of selection in sauropsid taxa in the *p53* network. This finding is similar to previous studies suggesting that upstream genes in metabolic pathways are targets of positive selection (Ramsay et al. 2009; McGaugh et al. 2015). Our data suggest that squamates (snakes and lizards) are likely driving this signature of upstream genes enriched for positive selection in the network. These findings are in agreement with a larger body of work that indicates that squamates have evolved differentially relative to other tetrapods (Castoe et al. 2009, 2013). Unique adaptations in this clade—such as tail regeneration in lizards (Alibardi 2016) and gut regression/regeneration in pythons (Andrew et al. 2017)—may promote this signature of positive selection in the *p53* network.

### Relation between Maximum Lifespan and *p53* Network Molecular Evolution

Senescence (mortality acceleration with advancing age), like cancer, is seen across the tree of multicellular life (Jones et al. 2014). Simply by living longer, species characterized by relatively longer lifespans should be at higher risk of disease due to accumulating somatic mutations (Gorbunova et al. 2014). Nonetheless, in nature, we find this is not the case as longer-lived organisms actually have lower incidences of age-related diseases (Peto et al. 1975). Decreased incidences of cancer in longer-lived species in nature have been associated with two nonmutually exclusive mechanisms. The first is copy number expansion in tumor suppressor genes. Although copy number expansion is generally detrimental (Hastings et al. 2009), copy number alterations can be beneficial as seen in longevity and cancer resistance in elephants (Abegglen et al. 2015) and super-*p53* transgenic mice (García-Cao et al. 2002). The second mechanism is increased selective pressures on genome maintenance systems that potentially reduce the accumulation of somatic mutations (Keane et al. 2015; MacRae et al. 2015), and thus can lead to longer lifespan (Jobson et al. 2010). Nonetheless, to date, a systematic analysis of the evolution of stress-response pathways across diverse taxa with diverse lifespans is lacking (but see: MacRae et al. 2015; McGaugh et al. 2015).

In this study, although we found little association between maximum lifespan and the number of genes with evidence of positive selection, we found a negative relationship between maximum lifespan and the number of genes with evidence of divergent molecular evolution (fig. 4). This means that species or lineages characterized by longer lifespans have fewer genes that vary significantly in their selection regimes relative to the rest of the tree, suggesting that both positive and purifying selection are driving this negative correlation (supplementary fig. S4, Supplementary Material online). Indeed, upstream genes with evidence of positive selection in the clade model tests were more commonly associated with species having shorter maximum lifespans (<30 years; supplementary fig. S4, Supplementary Material online), whereas genes with evidence of purifying selection relative to the rest of the tree were evenly distributed throughout the taxa.

Few comparative genomic aging studies to date have focused on longer-lived mammals (but see: Buffenstein 2005; Kim et al. 2011; Gorbunova et al. 2014), and even fewer still on sauropsids (but see: Shaffer et al. 2013; Reding et al. 2016). Yet these species may have diverse mechanisms associated with resistance to aging and thus age-related diseases (Buffenstein 2005). Taken together, our three lines of evidence suggest that species with longer maximum lifespans are more resistant to variation in molecular evolutionary forces—be they positive or purifying selection—that are divergent between mammals and sauropsids. Possible reasons for this include longer generation times over which molecular evolution can act such that the lack of divergent evolution is merely a by-product of longer lifespan. Alternatively, there may be constraints that impose limits to molecular changes overall due to the need for somatic maintenance during the extended reproductive lifespan of longer-lived species. Other studies have found an association between lifespan and positive selection in different stress-response pathways such as protective mechanisms associated with DNA repair (Kim et al. 2011) and inflammation (Fang et al. 2014).

In conclusion, comparative genomic analyses across a wide breadth of biodiversity can reveal shared and unique solutions to stress (McGaugh et al. 2015) and disease (Meadows and Lindblad-Toh 2017). Although some pathways may be highly conserved across taxa both in gene content and gene sequences, most pathways explored to date have diverged across diverse lineages (Tollis, Schiffman, et al. 2017). We find that overall, the *p53* network is enriched for genes with high divergence between mammals and sauropsids. Yet, the strength of both positive selection and divergent molecular evolution varied substantially across genes and taxa (mammals and sauropsids). We also identified variation in selective pressures in different portions of the network, driven predominantly by enrichment of significantly positively selected genes in squamates in the upstream portion of the network. Notably, we also found that longer-lived species have fewer genes with divergent molecular evolution (clade model C tests) among lineages, suggesting constraints in the modes of selection for species with longer lifespans in the *p53* network. In summary, our study extends comparative oncology studies, demonstrating evidence that comparative genomic approaches can provide insights into how networks, like the *p53* network, have evolved across diverse species and can lead to the identification of novel molecular targets for future treatments.

## Supplementary Material

Supplementary data are available at *Genome Biology and Evolution* online.

## Supplementary Material

Supplementary Data

## Notes

**Data deposition:** A majority of transcriptome and genome sequences were obtained from NCBI Sequence Read Archive. For 18 liver transcriptomes generated, the raw reads were deposited at Sequence Read Archive Study under the accessions SRA062458 and SRP017466. The Transcriptome assemblies, annotations, summaries and alignments were deposited in Dryad from a previous study and are available at dx.doi.org/10.5061.dryad.vn871.
