# Supplementary material for: Contrasting Patterns of Rapid Molecular Evolution within the p53 Network across Mammal and Sauropsid Lineages
Source: Genome Biol Evol. 2019 Jan 21;11(3):629–43. doi: 10.1093/gbe/evy273 (PMC6406535; doi:10.1093/gbe/evy273)
Supplement: Supplementary Data [file evy273_supp.zip › p53_supplement_GBE_Final.docx]

**Contrasting patterns of rapid molecular evolution within the *p53* network across mammal and sauropsid lineages.**

**Supplementary Material**

Courtney N. Passow, Anne M. Bronikowski, Heath Blackmon, Shikha Parsai, Tonia S. Schwartz, Suzanne E. McGaugh

**Materials and methods**

***Identification and clustering of p53 network genes***

Methods for the identification and clustering of *p53* network genes followed previously published protocols ([McGaugh, et al. 2015](#_ENREF_4)). For brevity, open reading frames from each transcriptome were clustered using USEARCH to reduce the number of isoforms and the centroid for each cluster was fed into program OrthoMCL. To identify putative orthologs, the program OrthoMCL ([Li, et al. 2003](#_ENREF_3)) was run on the USEARCH clustered transcriptomes and genome-derived gene sequences. OrthoMCL-defined orthologs often had multiple sequences per species, and so we again used the program USEARCH ([Edgar 2010](#_ENREF_1)) to create representative clusters within the OrthoMCL-clusters and filtered each cluster so that it contained only the single longest sequence per taxa per ortholog cluster ([Edgar 2010](#_ENREF_1)). We used the KEGG pathway for *p53*-signaling pathway ([Ogata, et al. 1999](#_ENREF_5)) to determine the focal genes in the *p53* network. To identify specific focal genes, we performed searches using Blastp (e-value cutoff of 1e-5; see methods) to find hits based on p53 KEGG-derived protein database. We also performed a second Blastp search using a UniProt database. For each gene, each sequence in the alignment exhibited a best blast hit to the same gene from the *p53* network for blasts to both of these databases (i.e. “focal genes”).

***Molecular evolutionary analyses in the p53 network***

To identify if there was exceptional variation in the *p53*-signaling network as compared to the rest of the genome, from our original alignments, we identified 1,414 putative orthologs that contained all 66 species and referred to them as “control genes”. The control genes served as a proxy for the remainder of the genome. Our dataset of focal genes contained most of the 66 species (mean = 57.8, median = 59, mode = 62). Note, control genes did not include frog, as they were not used to compare with the tests that included frog. The control genes in our study were ortholog clusters that contained sequences that were found in all 66 species and were potentially less divergent than genes where ortholog clusters were either split or where not every species was represented. Thus in comparisons between control and focal genes, we only included focal genes which formed one dominant cluster in OrthoMCL and USEARCH. We performed pairwise dN/dS comparisons to test between focal and control genes similar to a previous study ([McGaugh, et al. 2015](#_ENREF_4)).

***Statistical tests of molecular evolution with frog included***

We performed an additional analysis, which included sequences from frog (*Xenopus tropicalis*)*,* to polarize evolutionary changes along the branch leading to mammals and the branch leading to sauropsids. We used Ensembl homology search to identify one-to-one orthologs of frog with existing sequences in the alignments. Coding sequence for frog was imported into the previously aligned focal gene’s alignment and nucleotides were aligned posthoc in AliView 1.19 ([Larsson 2014](#_ENREF_2)) with the option “Realign everything as translated amino acids.” We then used the codeml program in PAML to identify evidence of positive selection and divergent selection between mammals and sauropsids and lineage-specific tests, with frog included. All analyses described in the main paper were then redone with the frog-inclusive PAML results.

***Weighted regression associated with lifespan and branch-site test and clade model C***

Weighted regressions were performed in R using the *lm* function ([Wilkinson and Rogers 1973](#_ENREF_6)). The number of genes that were found significant by either the clade model or branch-site tests in PAML was the response variables and the median of the reported maximum lifespans (Table S1). Because the number of species in each clade varied across clades, the count of genes from each clade was weighted by the inverse of the variance of maximum lifespans for taxa in each clade. Thus, clades that have greater variation in lifespan are down-weighted and provide less of a signal in the estimation of regression parameters, and taxa with little variation are weighted more heavily.

**Results**

***Centroid clustering***

Within the *p53*-signaling network, we were able to construct ortholog alignments with sufficient number of species for 45 genes. Of those, 12 of the 45 genes were split among separate USEARCH clusters after OrthoMCL analyses (*bax, bid, casp8, cdkn1a, fas, gtse1, mdm2, p48, p53, perp, serpine1*, and *shisa5*). In all cases, the 12 clusters were also split along multiple phylogenetic branches. The most extreme cases were *bid* (split in 9 mammal-only clusters, and 3 reptile-only clusters), *fas* (split into 3 mammal-only and 8 reptile-only clusters), *gste1* (split into 3 mammal-only and 4 reptile-only clusters), and *p53* (split into 1 mammal-only and 5 reptile-only clusters). Thus, these clusters were combined post-USEARCH and realigned into a single alignment per gene with only a single sequence representing each species (the longest was chosen). Since those 12 genes may be more divergent due to being manually-combined, they were excluded in the pairwise dN/dS comparisons to the control genes (which were from a dominant cluster from analyses with OrthoMCL). Several additional genes in the network were also split into multiple clusters (e.g. *apaf1, casp9, casp3, ccne1, chek2, gadd45g, mdm4, pidd*), however, each of these genes had a dominant cluster that contained sufficient species for analysis. Therefore, the clusters were not combined for analysis because the non-dominant clusters appeared to result from fragmented open-reading frames rather than from taxonomically-clustered sequences, as was the case for the manually-combined genes above.

***Network alignments and evolutionary rates in frogs***

When frog was included, we were only able to create alignments for 37 genes within the *p53* network, as only 37 genes had 1-to-1 orthologs with frog. The genes that were not included due to no 1-to-1 ortholog were *bid, casp8, casp9, ei24, igfbp3, sfn and shisa5.* Note, while one gene, *cdkn1a,* had an ortholog, it aligned poorly so it was not included in downstream analyses. Likewise, while *serpine1, rchy1, mdm4* and *mapk14* were included; all had poor regions within the alignment with frog. The number of species within alignments ranged from 47 – 67 per alignment (mean = 59.11, median = 60, mode = 65). When we performed the Chi-squared test to look at evidence of positive selection (branch-site test) and molecular evolutionary rates (clade model C) in upstream and downstream genes, we found some slight differences in the two analyses. For example, when frog was included, we lose significance in the reptile upstream and downstream interaction and the reptile and mammal upstream interaction (Table S10, S11). Likewise, we gain a new significant interaction with reptiles and mammals downstream (Table S11). While there are some slight differences, we still see a significant interaction in squamates *vs.* mammals upstream. Overall, results still allude to different rates of molecular evolution in genes upstream and downstream in the network within the two lineages, with squamates still being a predominate driver in reptiles (Table S10, S11). When we re-did the analyses focusing on the associations between lifespan and molecular evolution with frog included, we see the same results as before, where there was a significant negative correlation (*β =* -0.09*,* R^2^ = 0.50, p-value = 0.01) between lifespan and genes with evidence of divergent molecular evolution (clade model C; Figure S3). Interestingly, for alignments including frog, when we look at genes with evidence of positive selection and life span, we observe a slight significant interaction (*β =* 0.056*,* R^2^ = 0.08, p-value = 0.050), where there is a positive correlation between lifespan and genes under positive selection. However, while we see a slight significant trend, there seems to be no indication of what is driving this pattern (Figure S2). Hence, what might be driving some of these slight shifts may be the inability to include genes in the analysis due to issues of bad alignments and no available orthologs when frog was included in the tree.

**Supplemental Figures**


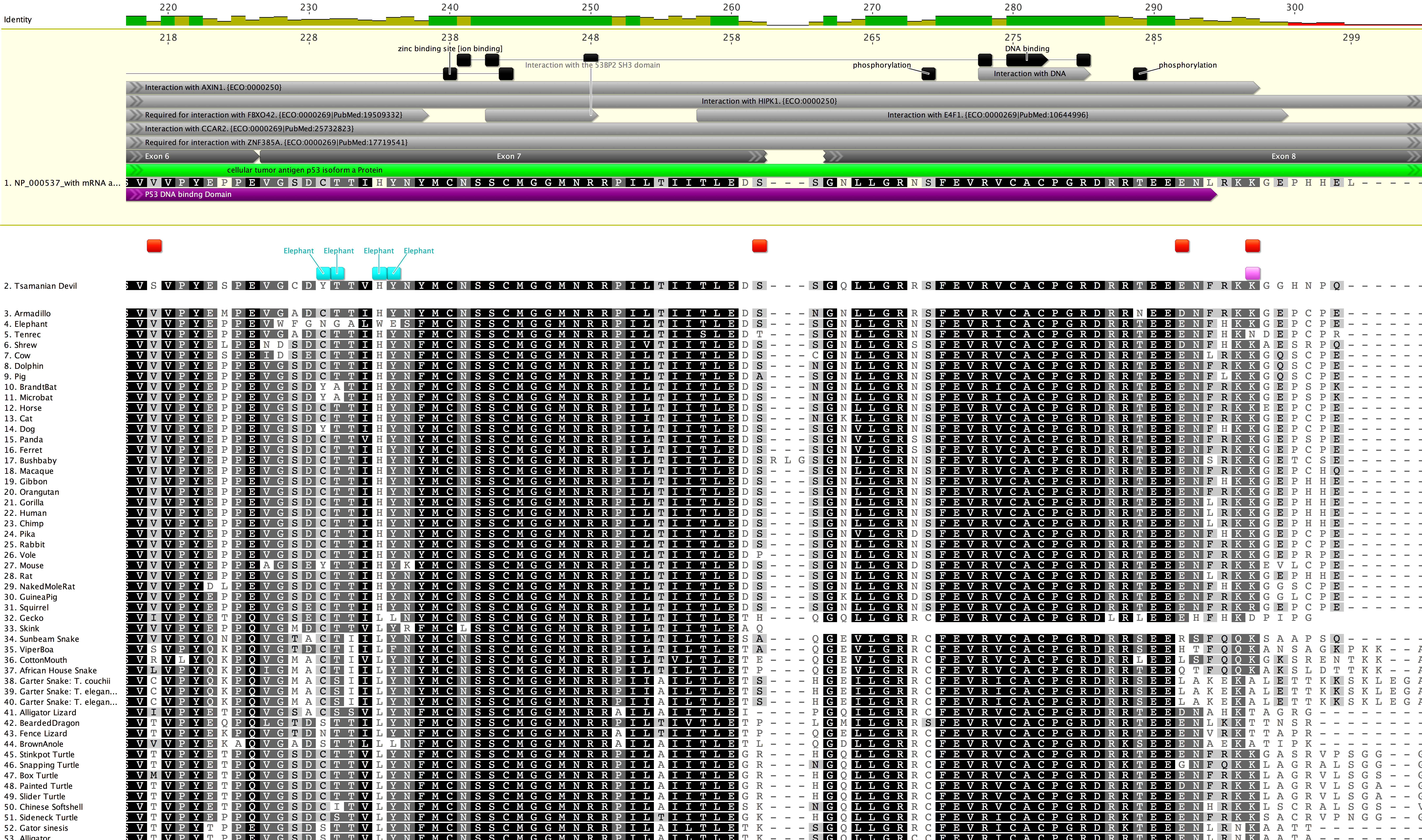

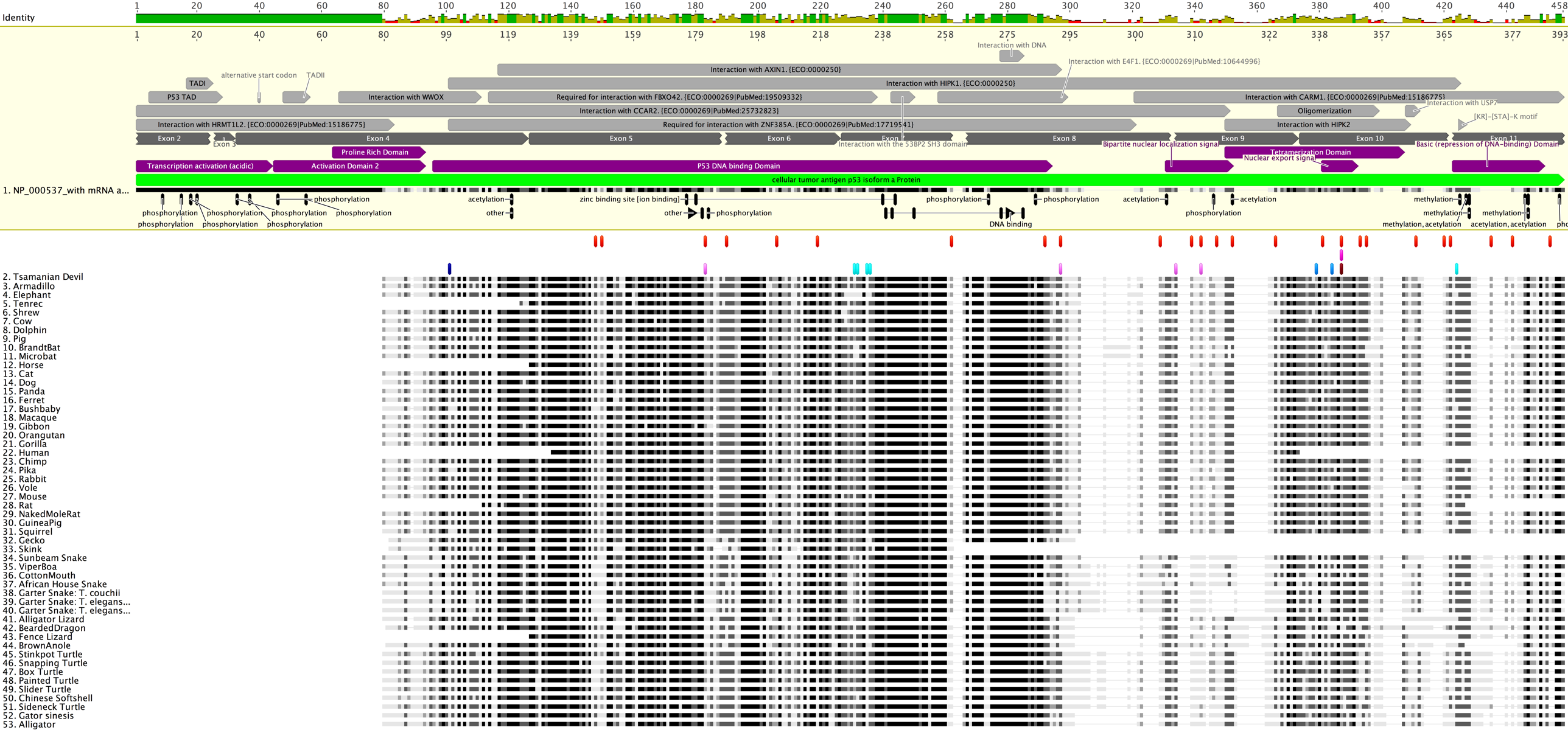


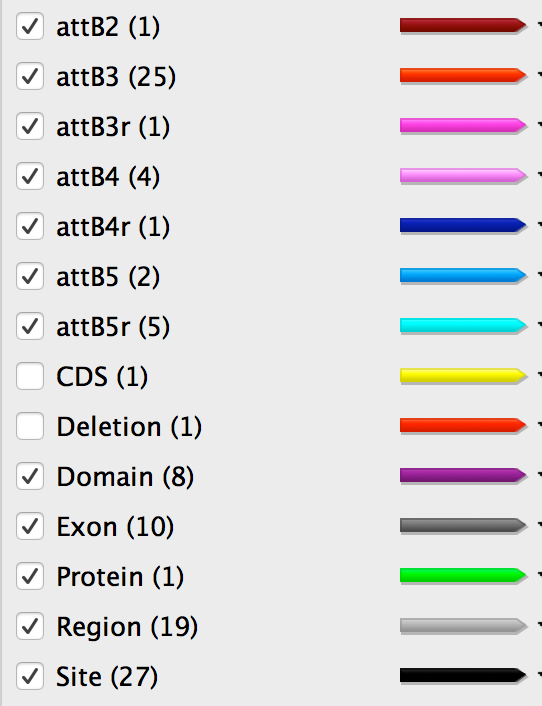


Reptile Branch

Reptile Clade

Squamates

Lizard

Mammal Branch

Mammal Clade

Elephant

**Sites with Evidence of**

**Positive Selection**


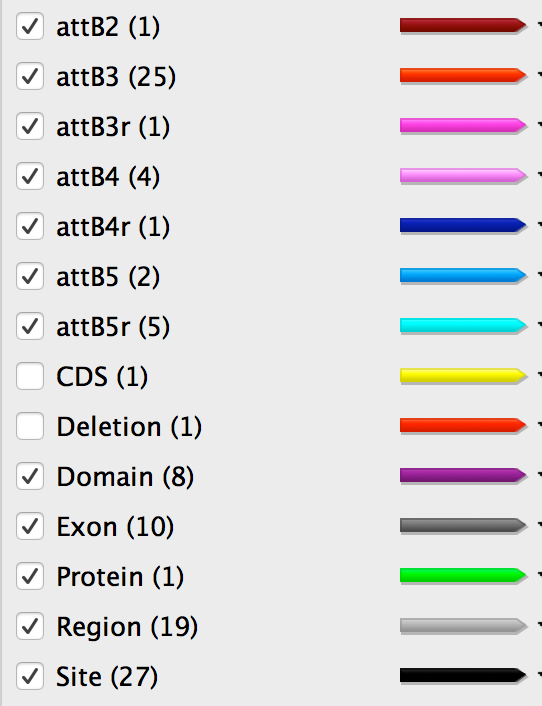


**General** **Annotations**

Domains

Exons

Protein Isoform

Regions

Modifications

**Annotation Legend**

**A.**

**B.**

**Figure S1.** P53 amino acid alignment against human reference (NP_000537) and annotations of sites under positive selection. Darker areas of the alignment indicate higher degree of similarity. The legend on the bottom right indicates the color-code for the annotations. **A.** Is the full p53 alignment; **B**. Represents a close up of the cluster of positively selected sites in the elephant.

**Figure S2. Correlation between longevity and genes under positive selection in the *p53* network when frogs are included. “**Sig. Gene Count” is the number of genes with significant evidence of positive selection via the branch-site test after sequential Bonferroni corrections. Lifespan is based on the median of the maximum lifespans (see Table S1) for all species used in lineage-specific comparisons.


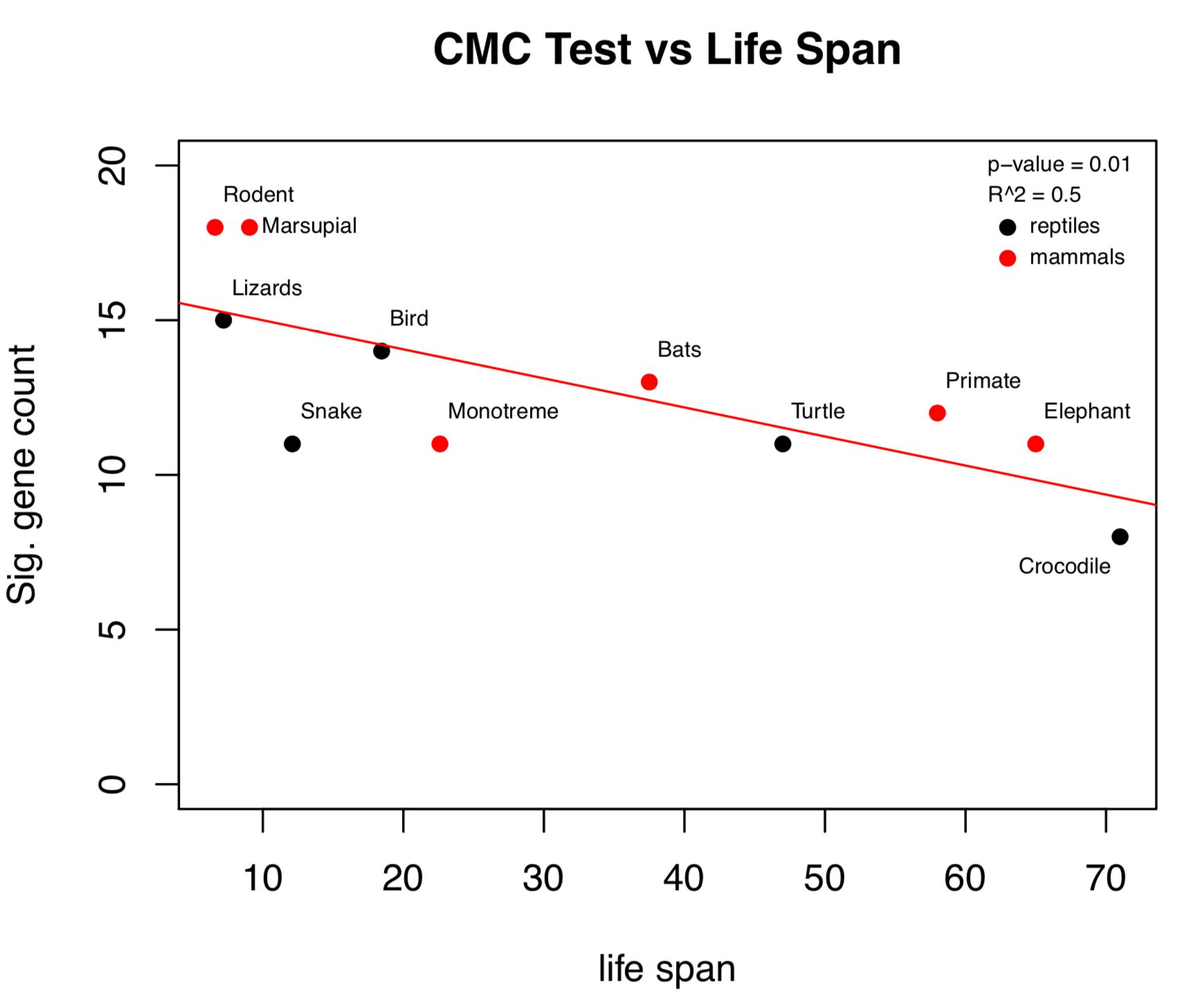


**Figure S3. Correlation between longevity and divergence in molecular evolution of the *p53* network when frog was included in the analyses. “**Sig. Gene Count” is the number of genes with significant evidence of divergent ω from the rest of the tree after sequential Bonferroni corrections for clade model C tests. Lifespan is based on the median of the maximum lifespans (see Table S1) for all species used in lineage-specific comparisons. This relationship is stronger than an analysis that did not include frog (Figure 4).


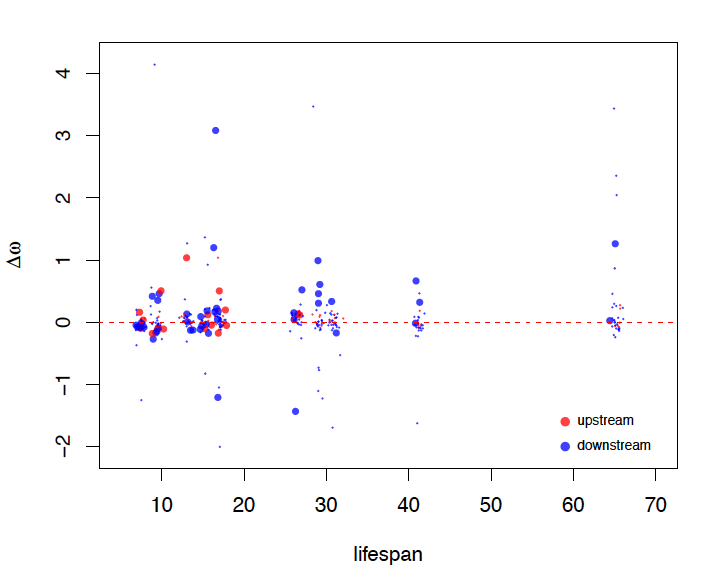


**Figure S4. Relationship between lifespan and** Δω **between focal clade and remainder of tree estimated with clade model C.** As clade model C tests for divergent evolutionary rates, the association between lifespan and ω could be due to various mixes of purifying and positive selection. To address this, we calculated Δω (differences in ω calculated for the focal (foreground) taxa relative to the background taxa in each clade model C test) and tested for correlations with lifespan. Generally, positive Δω indicates more positive selection in the foreground taxa, while negative values indicate more purifying selection in the foreground taxa. The size of the dot corresponds to significance, where larger dots are significant and smaller are not significant (after sequential Bonferroni corrections). We identified both positive and negative Δω scores driving significant clade model C tests within both upstream (red) and downstream (blue) genes, suggesting a mix of various types of selection on different portions of the *p53* network in mammals and reptiles. Interestingly, all upstream genes with significant Δω via the clade model were located in taxa with a median lifespan ≤ 30 years.

**Supplemental Tables**

**Table S1: Measures of longevity data collected from AnAge or the primary literature.** Reported are the maximum lifespan measures for each species used in the lineage-specific analyses. Longevity groups indicate what group each species was associated with in the PAML analyses. Longevity groups were selected for specific reptile and mammalian groups based on previous research, hence if there is no longevity group; it was not included in lineage-specific analyses. Note that for the longevity lineage-specific analyses, Chinese softshell turtle was not included as we were unable to find data for maximum lifespan for that species.

| **Species** | **Common name** | **Longevity group** | **Maximum lifespan** |
| --- | --- | --- | --- |
| *Ornithorhynchus anatinus* | Platypus | Monotreme | 22.6 |
| *Monodelphis domestica* | Opossum | Marsupial | 5.1 |
| *Sarcophilus harrisii* | Tasmanian devil | Marsupial | 13 |
| *Dasypus novemcinctus* | Armadillo |  | 22.3 |
| *Loxodonta africana* | Elephant | Elephant | 65 |
| *Echinops telfairi* | Tenrec |  | 19 |
| *Sorex araneus* | Shrew |  | 3.2 |
| *Bos taurus* | Cow |  | 20 |
| *Tursiops truncatus* | Dolphin |  | 51.6 |
| *Sus scrofa* | Pig |  | 27 |
| *Myotis brandtii* | Brandt bat | Bat | 41 |
| *Myotis lucifugus* | Little brown bat | Bat | 34 |
| *equus caballus* | Horse |  | 57 |
| *Felis catus* | Cat |  | 30 |
| *Canis lupus familiaris* | Dog |  | 24 |
| *Ailuropoda melanoleuca* | Panda |  | 36.8 |
| *Mustela putorius furo* | Ferret |  | 11.1 |
| *Otolemur garnettii* | Bushbaby | Primate | 20 |
| *Macaca mulatta* | Macaque | Primate | 40 |
| *Nomascus leucogenys* | White-cheeked gibbon | Primate | 44.1 |
| *Pongo abelii* | Orangutan | Primate | 58^a^ |
| *Gorilla gorilla* | Gorilla | Primate | 60.1 |
| *Homo sapien* | Human | Primate | 122.5 |
| *Pan troglodytes* | Chimp | Primate | 59.4 |
| *Ochotona princeps* | Pika |  | 7 |
| *Oryctolagus cuniculus* | European rabbit |  | 9 |
| *microtus ochrogaster* | Vole | Rodent | 5.3 |
| *Mus musculus* | Mouse | Rodent | 4 |
| *Rattus norvegicus* | Rat | Rodent | 3.8 |
| *Heterocephalus glaber* | Naked Mole Rat | Rodent | 31 |
| *Cavia porcellus* | Guinea Pig | Rodent | 12 |
| *Ictidomys tridecemlineatus* | Ground squirrel | Rodent | 7.9 |
| *Xenopeltis unicolor* | Sunbeam Snake | Snake | 12.1 |
| *Candoia aspera* | Viper Boa | Snake | 9.9 |
| *Agkistrodon piscivorus* | Cotton Mouth | Snake | 24.5 |
| *Lamprophis fuliginosus* | African House Snake | Snake | 9 |
| *Thamnophis couchii* | Garter Snake | Snake | 7.7 |
| *Thamnophis elegans* | Garter Snake - Lakeshore | Snake | 17.8 |
| *Thamnophis elegans* | Garter Snake - Meadow | Snake | 17.8 |
| *Elgaria multicarinata* | Alligator Lizard | Lizard | 9.8 |
| *Pogona vitticeps* | Bearded Dragon | Lizard | 12 |
| *Sceloporus undulatus* | Fence Lizard | Lizard | 4^b^ |
| *Anolis sagrei* | Brown Anole | Lizard | 6^c^ |
| *Anolis carolinensis* | Green Anole | Lizard | 7.2 |
| *Eublepharis macularius* | Leopard gecko | Lizard | 28.5 |
| *Scincella lateralis* | Ground skink | Lizard | 4^d^ |
| *Sternotherus odoratus* | Stinkpot turtle | Turtle | 26.6 |
| *Chelydra serpentina* | Snapping turtle | Turtle | 47 |
| *Chelonoidis nigra* | Galapagos tortoise | Turtle | 177 |
| *Terrapene carolina* | Box turtle | Turtle | 138 |
| *Chrysemys picta* | Painted turtle | Turtle | 61 |
| *Trachemys scripta* | Slider turtle | Turtle | 41.3 |
| *Pelodiscus sinensis* | Chinese softshell turtle | Turtle | NA |
| *Pelusios castaneus* | Sideneck turtle | Turtle | 47 |
| *Alligator sinensis* | Chinese Alligator | Crocodile | 65 |
| *Alligator mississippiensis* | Alligator | Crocodile | 77 |
| *Anas platyrhynchos* | Duck | Bird | 29.1 |
| *Gallus gallus* | Chicken | Bird | 30 |
| *Meleagris gallopavo* | Turkey | Bird | 13 |
| *Columba livia* | Pigeon | Bird | 35 |
| *Falco cherrug* | Saker falcon | Bird | 15.9 |
| *Falco peregrinus* | Peregrine falcon | Bird | 25 |
| *Melopsittacus undulatus* | Budgerigar | Bird | 21 |
| *Ficedula albicollis* | Collared flycatcher | Bird | 9.8 |
| *Geospiza fortis* | Darwin finch | Bird | 14^e^ |
| *Taeniopygia guttata* | Zebra finch | Bird | 12 |

^a^ Wich SA, Utami-Atmoko SS, Setia TM, Rijksen HD, Schürmann C, Van Hooff J, van Schaik CP 2004. Life history of wild Sumatran orangutans (*Pongo abelii*). Journal of human evolution 47: 385-398.

^b^ Beane JC, Braswell AL, Mitchell JC, Palmer WM. 2010. Amphibians and Reptiles of the Carolinas and Virginia: Univ of North Carolina Press.

^c^ Ferner JW 1976. Notes on natural history and behavior of *Sceloporus undulatus erythrocheilus* in Colorado. American Midland Naturalist: 291-302.

^d^ Mooi RD 2010. North American Wildlife: Cavendish Square Publishing.

^e^ Grant PR, Grant BR 1992. Demography and the genetically effective sizes of two populations of Darwin's finches. Ecology 73: 766-784.

**Table S2: Number of sequences for each gene in the *p53* network.** Each taxa is represented by the number of sequences from that group in the alignment. The number of individuals for squamates is the combination of snakes and lizards. Note that Eutherian mammals are part of the tree (Figure 1) and were part of the background for all calculations, however they were not included as a focal lineage-specific comparisons for the branch-site test or clade model C.

| **Gene** | **Eutherian mammals** | **Bats** | **Rodents** | **Elephants** | **Primates** | **Marsupials** | **Monotremes** | **Crocodilians** | | **Lizards** | **Birds** | **Turtles** | **Snakes** |
| --- | --- | --- | --- | --- | --- | --- | --- | --- | --- | --- | --- | --- | --- |
| APAF1 | 12 | 2 | 5 | 1 | 7 | 2 | 1 | 2 | 7 | | 10 | 8 | 7 |
| ATM | 13 | 2 | 6 | 1 | 7 | 2 | 1 | 2 | 7 | | 8 | 8 | 7 |
| ATR | 13 | 2 | 6 | 1 | 7 | 2 | 1 | 2 | 7 | | 10 | 7 | 7 |
| BAX | 13 | 1 | 6 | 1 | 7 | 1 | 0 | 2 | 5 | | 3 | 1 | 6 |
| BID | 10 | 2 | 6 | 1 | 6 | 2 | 1 | 2 | 7 | | 10 | 8 | 7 |
| CASP3 | 13 | 2 | 6 | 1 | 7 | 2 | 1 | 2 | 5 | | 10 | 7 | 4 |
| CASP8 | 13 | 2 | 6 | 1 | 7 | 2 | 1 | 2 | 0 | | 8 | 8 | 0 |
| CASP9 | 13 | 2 | 6 | 1 | 7 | 2 | 1 | 0 | 5 | | 9 | 4 | 0 |
| CCND1 | 10 | 2 | 6 | 1 | 7 | 2 | 1 | 2 | 7 | | 10 | 7 | 7 |
| CCND2 | 13 | 2 | 6 | 1 | 6 | 2 | 1 | 2 | 2 | | 10 | 7 | 0 |
| CCNE1 | 11 | 0 | 6 | 1 | 7 | 2 | 1 | 0 | 7 | | 8 | 8 | 7 |
| CCNG1 | 13 | 2 | 6 | 1 | 7 | 2 | 1 | 2 | 6 | | 10 | 8 | 4 |
| CDKN1A | 13 | 2 | 6 | 1 | 7 | 2 | 0 | 2 | 7 | | 9 | 6 | 7 |
| CHEK1 | 13 | 2 | 6 | 1 | 7 | 2 | 1 | 2 | 6 | | 10 | 7 | 5 |
| CHEK2 | 12 | 2 | 6 | 1 | 7 | 2 | 1 | 2 | 1 | | 10 | 7 | 0 |
| CYC | 11 | 2 | 5 | 1 | 7 | 1 | 1 | 2 | 6 | | 10 | 6 | 7 |
| EI24 | 13 | 2 | 6 | 1 | 7 | 2 | 1 | 2 | 7 | | 10 | 8 | 7 |
| FAS | 10 | 2 | 6 | 1 | 7 | 0 | 0 | 2 | 4 | | 10 | 8 | 4 |
| GADD45G | 13 | 1 | 6 | 1 | 7 | 2 | 0 | 2 | 4 | | 7 | 0 | 5 |
| GTSE1 | 10 | 0 | 6 | 1 | 7 | 0 | 0 | 1 | 4 | | 10 | 8 | 2 |
| IGF1 | 13 | 2 | 6 | 1 | 7 | 2 | 1 | 2 | 3 | | 10 | 5 | 6 |
| IGFBP3 | 12 | 0 | 6 | 1 | 7 | 2 | 1 | 2 | 5 | | 10 | 7 | 5 |
| MAPK14 | 13 | 2 | 5 | 1 | 6 | 2 | 0 | 2 | 7 | | 9 | 6 | 5 |
| MDM2 | 13 | 2 | 6 | 1 | 7 | 2 | 1 | 2 | 7 | | 10 | 8 | 7 |
| MDM4 | 13 | 2 | 6 | 1 | 7 | 2 | 1 | 1 | 5 | | 10 | 8 | 7 |
| P48 | 12 | 2 | 6 | 1 | 7 | 2 | 1 | 2 | 7 | | 9 | 8 | 5 |
| P53 | 13 | 2 | 6 | 1 | 7 | 1 | 0 | 2 | 6 | | 0 | 7 | 7 |
| P63 | 13 | 2 | 6 | 1 | 7 | 2 | 1 | 1 | 1 | | 10 | 3 | 0 |
| PERP | 13 | 2 | 6 | 1 | 7 | 1 | 1 | 2 | 7 | | 10 | 7 | 7 |
| PIDD | 10 | 0 | 6 | 1 | 5 | 0 | 0 | 2 | 6 | | 10 | 7 | 6 |
| PPM1D | 13 | 2 | 6 | 1 | 7 | 2 | 1 | 2 | 7 | | 10 | 8 | 6 |
| PTEN | 13 | 2 | 6 | 1 | 7 | 2 | 1 | 2 | 7 | | 10 | 8 | 7 |
| RCHY1 | 12 | 2 | 6 | 1 | 7 | 2 | 1 | 2 | 2 | | 10 | 7 | 0 |
| RFWD2 | 13 | 2 | 6 | 1 | 7 | 2 | 1 | 2 | 7 | | 10 | 8 | 7 |
| RRM2b | 12 | 2 | 5 | 1 | 7 | 2 | 1 | 1 | 6 | | 9 | 6 | 6 |
| SERPINb5 | 13 | 2 | 6 | 1 | 7 | 2 | 1 | 1 | 0 | | 9 | 5 | 0 |
| SERPINE1 | 13 | 2 | 6 | 1 | 7 | 2 | 1 | 1 | 5 | | 0 | 7 | 4 |
| SESN3 | 13 | 2 | 6 | 1 | 7 | 2 | 1 | 1 | 6 | | 10 | 7 | 6 |
| SFN | 13 | 2 | 6 | 1 | 7 | 1 | 1 | 1 | 1 | | 10 | 2 | 0 |
| SHISA5 | 11 | 0 | 6 | 1 | 7 | 1 | 1 | 1 | 7 | | 9 | 8 | 7 |
| SIAH1 | 12 | 2 | 6 | 1 | 7 | 2 | 0 | 2 | 7 | | 10 | 8 | 7 |
| SIRT6 | 10 | 2 | 5 | 1 | 6 | 2 | 1 | 2 | 6 | | 10 | 8 | 7 |
| STEAP3 | 13 | 2 | 6 | 1 | 6 | 2 | 0 | 2 | 7 | | 10 | 8 | 7 |
| TSC2 | 13 | 2 | 6 | 1 | 7 | 2 | 1 | 2 | 7 | | 10 | 8 | 7 |
| ZMAT3 | 13 | 2 | 6 | 1 | 7 | 2 | 1 | 2 | 3 | | 10 | 4 | 2 |

**Table S3. Estimates of sequence variation between sauropsids and mammals.** Total is the number of sequences in the alignment, N is the total number of pairwise comparisons between sauropsids and mammals used to calculate measures D_N_ (nonsynonymous substitutions per nonsynonymous sites); D_S_ (synonymous substitutions per synonymous sites); and ω (dN/dS) are medians from the pairwise comparisons calculated in PAML between sauropsids and mammals. Italicized and bolded symbols are genes for which alignments were made by combining centroids (see methods and supplementary material) and were thus excluded from pairwise dN/dS comparisons to control genes (described in supplementary material), as these are likely very divergent genes.

| **Gene** | **Total** | **N** | **D_N_** | **D_S_** | **ω** |
| --- | --- | --- | --- | --- | --- |
| APAF1 | 64 | 1016 | 0.19 | 1.43 | 0.13 |
| ATM | 64 | 949 | 0.16 | 1.53 | 0.10 |
| ATR | 65 | 1023 | 0.09 | 1.41 | 0.06 |
| ***BAX*** | 46 | 493 | 0.22 | 2.04 | 0.11 |
| ***BID*** | 62 | 952 | 0.59 | 2.53 | 0.24 |
| CASP3 | 60 | 896 | 0.24 | 1.72 | 0.14 |
| ***CASP8*** | 50 | 576 | 0.36 | 1.69 | 0.21 |
| CASP9 | 50 | 576 | 0.26 | 1.62 | 0.16 |
| CCND1 | 62 | 957 | 0.09 | 3.00 | 0.03 |
| CCND2 | 52 | 651 | 0.08 | 1.48 | 0.05 |
| CCNE1 | 58 | 840 | 0.17 | 1.77 | 0.10 |
| CCNG1 | 62 | 960 | 0.12 | 1.61 | 0.07 |
| ***CDKN1A*** | 62 | 961 | 0.60 | 2.48 | 0.24 |
| CHEK1 | 62 | 960 | 0.11 | 3.00 | 0.04 |
| CHEK2 | 51 | 620 | 0.20 | 1.35 | 0.15 |
| CYC | 59 | 868 | 0.06 | 1.62 | 0.04 |
| EI24 | 66 | 1088 | 0.06 | 2.35 | 0.02 |
| ***FAS*** | 54 | 728 | 0.58 | 2.74 | 0.22 |
| GADD45G | 48 | 540 | 0.12 | 1.52 | 0.08 |
| ***GTSE1*** | 49 | 600 | 0.65 | 1.94 | 0.34 |
| IGF1 | 58 | 832 | 0.17 | 1.30 | 0.12 |
| IGFBP3 | 58 | 841 | 0.14 | 3.00 | 0.05 |
| MAPK14 | 58 | 841 | 0.03 | 1.04 | 0.03 |
| ***MDM2*** | 66 | 1084 | 0.24 | 1.47 | 0.16 |
| MDM4 | 63 | 992 | 0.29 | 1.86 | 0.15 |
| ***P48*** | 62 | 961 | 0.28 | 1.81 | 0.15 |
| ***P53*** | 52 | 660 | 0.38 | 1.83 | 0.22 |
| P63 | 47 | 480 | 0.03 | 1.11 | 0.02 |
| ***PERP*** | 64 | 1023 | 0.39 | 1.45 | 0.27 |
| PIDD | 53 | 682 | 0.35 | 1.59 | 0.22 |
| PPM1D | 65 | 1056 | 0.16 | 2.17 | 0.08 |
| PTEN | 66 | 1054 | 0.03 | 0.57 | 0.05 |
| RCHY1 | 52 | 649 | 0.10 | 2.06 | 0.05 |
| RFWD2 | 66 | 1085 | 0.01 | 1.42 | 0.01 |
| RRM2b | 58 | 840 | 0.05 | 1.49 | 0.04 |
| SERPINb5 | 47 | 480 | 0.23 | 1.60 | 0.14 |
| ***SERPINE1*** | 49 | 544 | 0.30 | 1.11 | 0.27 |
| SESN3 | 62 | 960 | 0.05 | 1.11 | 0.05 |
| SFN | 45 | 434 | 0.16 | 0.70 | 0.23 |
| ***SHISA5*** | 59 | 864 | 0.24 | 3.00 | 0.11 |
| SIAH1 | 64 | 1020 | 0.00 | 0.81 | 0.00 |
| SIRT6 | 60 | 891 | 0.18 | 1.28 | 0.14 |
| STEAP3 | 64 | 1020 | 0.20 | 1.41 | 0.14 |
| TSC2 | 66 | 1061 | 0.11 | 1.83 | 0.06 |
| ZMAT3 | 53 | 672 | 0.11 | 1.48 | 0.07 |

**Table S4:** **Estimates of sequence divergence between focal (*p53* network genes) and control (representative of genome) between mammals and sauropsids.** We report control and focal genes, comparisons, focal gene names, whether it is a control or focal gene, number of sequences in the dN/dS comparison, and median value for dN/dS, dN and dS only. Due to the size of the document we provide it as an excel sheet termed – *Table S4 DN/DS comparison.xlsx.* Note that genes where alignments were made by combining multiple clusters (noted in Table S3) were not included in this comparison.

**Table S5. Results from branch-site tests for positive selection on *p53* network genes when whole sauropsids and mammal clades are placed in the foreground.** Chi-square values are given from likelihood ratio tests from PAML. Bold values were significant at p < 0.05 prior to sequential Bonferroni correction. An asterisk signifies p < 0.05 after sequential Bonferroni correction. Significant values suggest evidence for positive selection for the specified phylogenetic clade at some sites within the alignment.

| **Gene** | **Mammal clade** | **Sauropsids clade** |
| --- | --- | --- |
| APAF1 | 0 | 0 |
| ATM | 0 | 0 |
| ATR | 0 | 0 |
| BAX | 0 | 0 |
| BID | **16.99*** | **32.25*** |
| CASP3 | 0 | 0 |
| CASP8 | **4.75** | 0 |
| CASP9 | -46.85 | -19.41 |
| CCND1 | 0 | -0.82 |
| CCND2 | 0 | -16.95 |
| CCNE1 | 0 | 0 |
| CCNG1 | 0 | 0 |
| CDKN1A | 0 | 0 |
| CHEK1 | 0 | 0 |
| CHEK2 | 0 | 0 |
| CYC | 0 | 0 |
| EI24 | 0 | 0 |
| FAS | **143.82*** | 0 |
| GADD45G | 0 | 0 |
| GTSE1 | -2.54 | 0.18 |
| IGF1 | **11.30*** | 0 |
| IGFBP3 | 0 | 0 |
| MAPK14 | 0 | 0 |
| MDM2 | 0 | 0 |
| MDM4 | 0 | 0 |
| P48 | 0 | 0 |
| P53 | 0 | 0 |
| P63 | -2.46 | 0 |
| PERP | -53.97 | 0 |
| PIDD | 0 | 0 |
| PPM1D | 0 | 0 |
| PTEN | -0.57 | 0 |
| RCHY1 | 0 | 0 |
| RFWD2 | 0 | 0.01 |
| RRM2b | 0 | 0 |
| SERPINB5 | 0 | 0 |
| SERPINE1 | 0 | 0 |
| SESN3 | 0 | -26.89 |
| SFN | **142.86*** | 0 |
| SHISA5 | -5.17 | 0 |
| SIAH1 | -0.02 | 0 |
| SIRT6 | 0 | 0 |
| Steap3 | 0 | 0 |
| TSC2 | -364.11 | 0 |
| ZMAT3 | 0 | 0 |

**Table S6. Results from branch-site tests for positive selection on *p53* network genes in sauropsids and mammals when ancestral branches are placed in the foreground.** Chi-square values are given from likelihood ratio tests from PAML. Bold values are significant at p < 0.05 prior to sequential Bonferroni correction. An asterisk signifies p < 0.05 after sequential Bonferroni correction. Significant values suggest evidence for positive selection for the specified phylogenetic branch at some sites within the alignment. All branch-site tests used the branch leading to the specific taxa as the foreground branch. NA’s represent no sequence for that gene in that foreground branch. Due to the size of the document we provide it as an excel sheet termed – *Table S6 Branch-site.xlsx.*

**Table S7. Results from branch-site tests for positive selection on *p53* network genes in sauropsids and mammals when ancestral branches are placed in the foreground and when frog is included.** Chi-square values are given from likelihood ratio tests from PAML. Bold values are significant at p < 0.05 prior to sequential Bonferroni correction. An asterisk signifies p < 0.05 after sequential Bonferroni correction. Significant values suggest evidence for positive selection for the specified phylogenetic branch at some sites within the alignment. All branch-site tests used the branch leading to the specific taxa as the foreground branch. NA’s represent no sequence for that gene in that foreground branch. Due to the size of the document we provide it as an excel sheet termed – *Table S7 Branch-site with frog.xlsx.*

**Table S8: Results from clade model tests for divergent dN/dS for *p53* network genes in sauropsids and mammals when different clades are placed in the foreground.** Chi-square values from likelihood ratio tests from PAML, where significant values suggest evidence of divergent selection for a set of sites between the foreground clade and the remainder of the tree. Bold values are significant at p < 0.05 prior to sequential Bonferroni correction. An asterisk signifies p < 0.05 after sequential Bonferroni correction. NA’s equal no sequence for that gene in a specific foreground branch. Due to the size of the document we provide it as an excel sheet termed – *Table S8 clade model C.xlsx.*

**Table S9: Results from clade model tests for divergent dN/dS for *p53* network genes in sauropsids and mammals when different clades are placed in the foreground and when frog is included.** Chi-square values from likelihood ratio tests from PAML, where significant values suggest evidence of divergent selection for a set of sites between the foreground clade and the remainder of the tree. Bold values are significant at p < 0.05 prior to sequential Bonferroni correction. An asterisk signifies p < 0.05 after sequential Bonferroni correction. NA’s equal no sequence for that gene in a specific foreground branch. Due to the size of the document we provide it as an excel sheet termed – *Table S9 clade model C with frog.xlsx.*

**Table S10: Upstream versus downstream gene input data and results of chi-squared tests:** Chi-squared tests were run to determine whether the number of genes identified with the branch-site tests and clade model differed within upstream and downstream genes and within or among clades. Because sample sizes for some cells in the contingency tables are less than five we calculated p-values using 2000 Monte Carlo simulations.

|  | Clades/Groupings | Significant | Not significant | χ*^2^* | p-value |
| --- | --- | --- | --- | --- | --- |
| Branch-site | Mammal upstream | 4 | 54 |  |  |
|  | Mammal downstream | 16 | 200 | 0.0176 | 1.0000 |
|  | Sauropsid upstream | 10 | 37 |  |  |
|  | Sauropsid downstream | 14 | 154 | 6.2042 | **0.0245** |
|  | Mammal upstream | 4 | 54 |  |  |
|  | Sauropsid upstream | 10 | 37 | 4.6459 | **0.0465** |
|  | Mammal downstream | 16 | 200 |  |  |
|  | Sauropsid downstream | 14 | 154 | 0.1125 | 0.8336 |
|  | Squamate upstream | 6 | 12 |  |  |
|  | Mammal upstream | 5 | 54 | 6.9605 | **0.0155** |
| Clade model C | Mammal upstream | 15 | 43 |  |  |
|  | Mammal downstream | 35 | 181 | 2.8590 | 0.1174 |
|  | Sauropsid upstream | 9 | 38 |  |  |
|  | Sauropsid downstream | 25 | 143 | 0.5025 | 0.4983 |

**Table S11: Upstream versus downstream gene input data and results of chi-squared tests for frog-inclusive analyses:** Chi-squared tests were run to determine whether the number of genes identified with the branch-site tests and clade model differed for upstream and downstream genes and within or among clades. Because sample sizes for some cells in the contingency tables are less than five we calculated p-values using 2000 Monte Carlo simulations.

|  | Clades/Groupings | Significant | Not significant | χ*^2^* | p-value |
| --- | --- | --- | --- | --- | --- |
| Branch-site | Mammal upstream | 8 | 50 |  |  |
|  | Mammal downstream | 17 | 138 | 0.325 | 0.642 |
|  | Sauropsid upstream | 12 | 35 |  |  |
|  | Sauropsid downstream | 26 | 107 | 0.746 | 0.412 |
|  | Mammal upstream | 8 | 50 |  |  |
|  | Sauropsid upstream | 12 | 35 | 2.320 | 0.153 |
|  | Mammal downstream | 26 | 107 |  |  |
|  | Sauropsid downstream | 17 | 138 | 4.150 | **0.046** |
|  | Squamate upstream | 7 | 13 |  |  |
|  | Mammal upstream | 8 | 50 | 4.306 | **0.047** |
| Clade model C | Mammal upstream | 28 | 30 |  |  |
|  | Mammal downstream | 55 | 100 | 2.904 | 0.125 |
|  | Sauropsid upstream | 17 | 30 |  |  |
|  | Sauropsid downstream | 42 | 91 | 0.332 | 0.603 |

**Table S12: Comparison of weighted regression to the unweighted regression.** Unweighted regressions were compared with regressions where the data was weighted by the inverse of the variance or standard error of the mean of the maximum life span for species in each lineage.

|  |  | **Weighting variable** | | | | |
| --- | --- | --- | --- | --- | --- | --- |
| **Formula** |  | **none** | | **1/var** | | **1/SE** |
| LS~BS | coef | 0.0927 | 0.0759 | | 0.0468 | |
|  | p-val | 0.1258 | 0.0013 | | 0.1554 | |
|  | Rsquared | 0.1560 | 0.6661 | | 0.1232 | |
| LS~CMC | coef | -0.1159 | -0.1261 | | -0.0913 | |
|  | p-val | 0.0147 | 0.0002 | | 0.0069 | |
|  | Rsquared | 0.4248 | 0.7688 | | 0.5262 | |

**References**

Edgar RC 2010. Search and clustering orders of magnitude faster than BLAST. Bioinformatics. 26: 2460-2461.

Larsson A 2014. AliView: a fast and lightweight alignment viewer and editor for large datasets. Bioinformatics. 30: 3276-3278.

Li L, Stoeckert CJ, Roos DS 2003. OrthoMCL: identification of ortholog groups for eukaryotic genomes. Genome research. 13: 2178-2189.

McGaugh SE, Bronikowski AM, Kuo C-H, Reding DM, Addis EA, Flagel LE, Janzen FJ, Schwartz TS 2015. Rapid molecular evolution across amniotes of the IIS/TOR network. PNAS. 112: 7055-7060.

Ogata H, Goto S, Sato K, Fujibuchi W, Bono H, Kanehisa M 1999. KEGG: Kyoto encyclopedia of genes and genomes. Nucleic Acid Res. 27: 29-34.

Wilkinson G, Rogers C 1973. Symbolic description of factorial models for analysis of variance. Applied Statistics: 392-399.
